# Fourier Spectral Deconvolution to Describe Behaviors of pH-Dependent Monomeric and Self-Associated Anthocyanin Species

**DOI:** 10.1021/acs.jcim.4c02300

**Published:** 2025-03-03

**Authors:** Rachael A. Tindal, David W. Jeffery, Richard A. Muhlack

**Affiliations:** Australian Research Council Training Centre for Innovative Wine Production and Waite Research Institute, The University of Adelaide, PMB 1, Glen Osmond, South Australia 5064, Australia

## Abstract

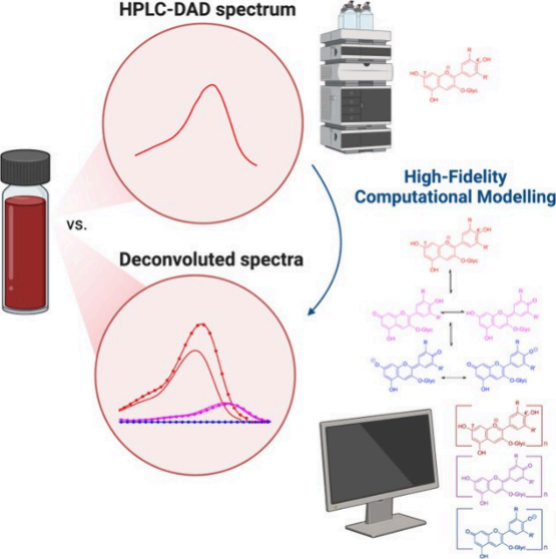

Anthocyanins are pigmented polyphenolic compounds that influence the color, stability, and quality of numerous plant organs (e.g., fruit, flowers) and their derived products (e.g., natural dyes, red wine). Existing within a complex multistate system, anthocyanins can be simultaneously present as pH-dependent red, purple, or blue species that are either in a monomeric (chemically unstable) or in a self-associated (temporally stable) form. However, limitations of current analytical techniques (e.g., HPLC with a UV–vis detector) may cause experimental data to omit or misrepresent important color and stability characteristics afforded by all anthocyanin species within the natural matrix. In response, a computational spectral deconvolution method is demonstrated that increases the fidelity of spectral data collected for anthocyanins, thereby representing all existing monomeric and self-associated anthocyanin species within solution for the first time. Case studies for the developed deconvolution model are presented, based on experimental data obtained via HPLC-DAD analysis for malvidin-3-*O*-β-d-glucopyranoside (M3G) in red wines that were sampled throughout fermentation. Fourier spectral deconvolution methods were used to transform experimental spectra for pigmented anthocyanin monomers into systems that represent spectral behaviors of all pigmented monomeric and self-associated anthocyanin species in solution. The developed computational model was found to significantly increase the level of signal feature extraction for the spectral data of anthocyanins, providing key information on color expression and stability characteristics that would be otherwise unattainable with traditional approaches using HPLC with UV–vis detection. The current work increases understanding and control over key attributes of anthocyanins and has broad potential applications for the analysis and commercialization of anthocyanin-containing products.

## 1 Introduction

Anthocyanins are polyphenolic compounds that provide red, purple, and blue hues to plant organs including fruits (e.g., malvidin from grape berries, delphinidin from pomegranates), flowers (cyanidin from roses, peonidin from peony flowers), and leaves (e.g., cyanidin from autumn leaves) across the plant kingdom.^1−5^ Being stabilized in glycosidic form, anthocyanins can be applied for use as natural pigments in red wines, foods, beverages, dyes, textiles, cosmetics, and pH-sensors,^6−9,4,10^ where their color is a key factor that influences product quality and consumer preferences.^11−13^

**Figure 1:**
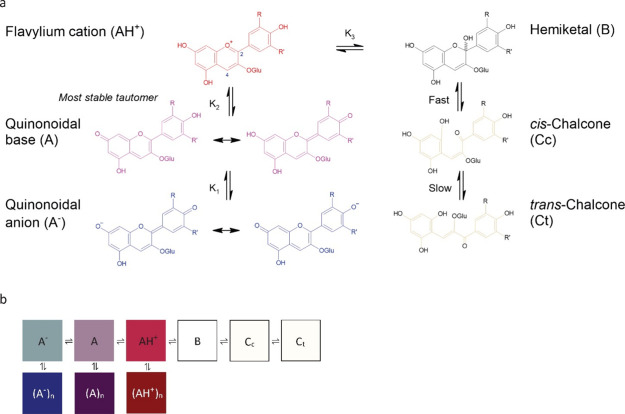
(a) Structures for pH-dependent transformations between monomeric anthocyanins, and (b) a scheme of pH-dependent transformations between monomeric anthocyanins (horizontal) with color-enhancing self-association interactions between same-species monomers (vertical). Adapted from Tindal et al.^46^ CC BY http://creativecommons.org/licenses/by/4.0/.

Anthocyanins exist within a complex pH-dependent multistate system of monomeric and self-associated species, with the latter exhibiting greater temporal stability. The highly reactive monomers undergo acid–base reactions, generating the red flavylium cation (AH^+^), purple quinonoidal base (A), and blue quinonoidal anion (A^–^), a hydration reaction that produces the colorless hemiketal (i.e., pseudobase, B),^14,15^ and tautomerisation and isomerization reactions that generate the pale yellow *cis*- and *trans*- chalcone (*Cc* and Ct) species (Figure 1a). The multistate is dependent on pH such that the flavylium cation dominates in acidic conditions, the quinonoidal base and hemiketal at neutral pH values, and the quinonoidal anion in alkaline media.^16,17^ Self-association is a key mechanism that facilitates anthocyanin color stabilization, whereby pigmented monomers of the same-species undergo physical π–π stacking, which inhibits hemiketal formation.^18,16^ Resultant self-associated species are the dark red flavylium cation (AH^+^)_*n*_, dark purple quinonoidal base (A)_*n*_, and dark blue quinonoidal anion (A^–^)_*n*_, where *n* indicates the degree of self-association according to the number of monomers involved^19,20^ (Figure 1b). Self-association may also increase the likelihood of other color-stabilizing reactions occurring in solution over time (e.g., intermolecular copigmentation, polymerization) as it preserves pigmented anthocyanin monomers.^21−23^

High-performance liquid chromatography with diode array detection (HPLC-DAD) methods are typically used to quantify anthocyanin concentration and color, where the pH-controlled eluent required by HPLC can stabilize chemically and physically reactive pH-dependent monomers.^16,24,25,20,26^ Typically, an acidic eluent (pH ≤ 2.50) is used to fix anthocyanins in their red monomeric flavylium cation form (AH^+^),^27,16,17^ as this allows for optimal detection and quantification of anthocyanins (e.g., maximum absorbance in the visible region at 520 nm) and minimizes tailing and broadening of chromatographic peaks.^28,1,29^ In other instances, neutral and basic HPLC eluents are chosen to quantify anthocyanins in their monomeric purple quinonoidal base (i.e., A) or blue quinonoidal anion (i.e., A^–^) forms, respectively;^29,1^ although, these are less commonly used, since A and A^–^ are relatively unstable and susceptible to hemiketal formation during analysis.^14^ However, HPLC eluents can interfere with Vis spectral data acquisition and potentially lead to misrepresentative results, since they force anthocyanins to be fixed as single pH-dependent species (i.e., with λ_max_ values of approximately 520 nm for AH^+^, 570 nm for A, 620 nm for A^–^)^30^ and thereby fail to represent all coexisting pigmented species (i.e., AH^+^, A, A^–^, (AH^+^)_*n*_, (A)_*n*_, (A^–^)_*n*_) that provide essential color and stability attributes.^20,16,30,31,26^ The analytical limitations have significant consequences for the characterization and development of anthocyanin-containing products (e.g., dyes, pH-sensors, red wines), as spectral data is a crucial and frequently used tool for anthocyanin characterization in research and industry settings.^1,32,33^ Current analytical methods must be refined in order to improve the accuracy of collected data and afford researchers with increased control over the complex anthocyanin multistate system.

Fourier spectral deconvolution (FSD) is a powerful computational tool that can overcome the aforementioned shortcomings by enhancing the fidelity, accuracy and level of signal feature extraction for multiple types of spectra. Used originally for signal and image processing and applied more recently to the analysis of biological and chemical data,^34−36^ the process of deconvolution combines spectral data with additional properties of the considered system, revealing more detailed characteristics of the original spectra.^37^ To address the evident challenges, the current study has developed a multiphase deconvolution model that allows for a spectrum representing a single pigmented monomeric species to be transformed into a system of spectral data that represents all pigmented monomeric and self-associated anthocyanin species. The individual spectrum undergoes preprocessing operations to reduce noise and is then incorporated with experimentally validated chemical parameters corresponding to the sample and HPLC-DAD eluent conditions. Following computational deconvolution methods, new and accurate spectral data for all pigmented anthocyanin species are constructed. In total, the current work aims for the first time to increase the fidelity of data describing anthocyanins during chemical analysis, thereby addressing significant analytical limitations and strengthening opportunities for the development of anthocyanin-containing products.

## 2 Materials and Methods

### 2.1 Experimental Method

#### 2.1.1 Sample Preparation

Experimental data for malvidin-3-*O*-β-d-glucopyranoside (M3G) in red wines was obtained to conduct case studies for the developed deconvolution model. M3G was chosen as the model anthocyanin as it is readily available as an analytical standard, as well as being the dominant anthocyanin in wine.^38,26^ Wine samples were collected daily throughout fermentation under large-scale commercial conditions at Pernod Ricard Winemakers in the Barossa Valley. Sample collection was completed in triplicate for three Shiraz ferments (W_1_, W_2_, W_3_) from tanks with respective volumes of 175, 44, and 16 kL. Samples were collected approximately every 24–48 h throughout fermentation, with fermentation durations determined to be 196.8, 273.5, and 284.0 h, respectively. Samples were frozen upon collection and stored at −20 °C until analysis. Values for sample pH and ferment temperature were obtained from empirical winery analysis.

After thawing at room temperature, 300 μL of each wine sample was diluted into 1200 μL of a solution containing 1.5% phosphoric acid, 1.0% acetonitrile, and 97.5% Milli-Q ultrapure water. Samples were mixed for 10 s using a Ratek vortex mixer (Adelab 97 Scientific, Thebarton, SA, Australia), centrifuged for 10 min at 6010*g*, and transferred into 2 mL amber HPLC vials. Calibration solutions were prepared for HPLC analysis through dilution with Milli-Q ultrapure water of M3G (≥95% by HPLC, Extrasynthese, Genay, France) stock solution.

#### 2.1.2 Quantification of M3G

Red wine samples underwent HPLC analysis using an Agilent 1100 Series Instrument (Agilent, Forest Hill, VIC, Australia) with a diode array detector (DAD) and quaternary pump. Conditions for gradient elution were based on methods by Cozzolino et al.^39^ and Mercurio et al.,^40^ with an injection volume of 20 μL. The acidic eluent containing phosphoric acid and acetonitrile possessed a pH of 2.50, which accounts for the presence of organic solvent.^41,42^ Instrument control and data analysis were conducted with Agilent ChemStation software (B.04.03 SP1), and M3G was quantified at 280 and 520 nm using external calibration. Anthocyanin absorbance spectra were recorded between 210 and 600 at 1 nm intervals, with a path length of 10 mm and examined at 520 nm, in accordance with standard red wine analytical methods.^32,33,29^

### 2.2 Computational Method

**Figure 2:**
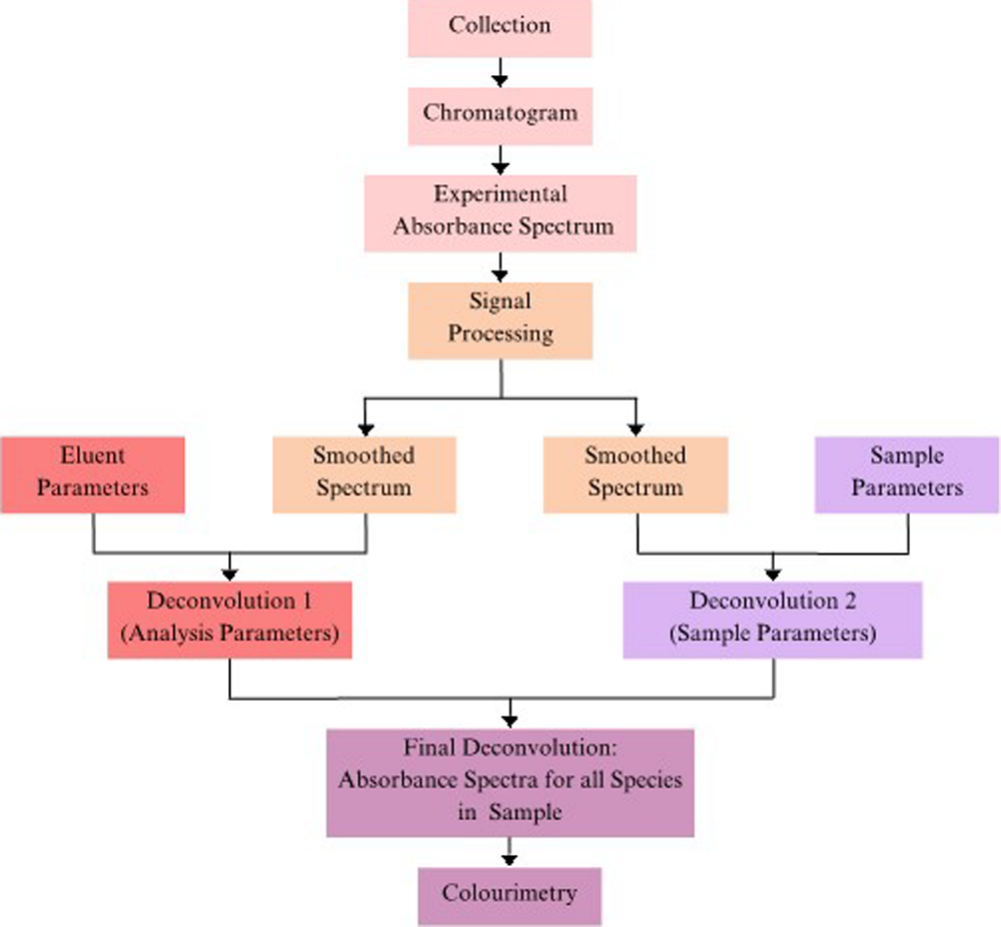
A workflow diagram representing the process of the developed deconvolution method, as outlined in the following steps. Samples are collected and analyzed to obtain an experimental chromatogram and absorbance spectrum for an anthocyanin (e.g., M3G). Each spectrum undergoes signal processing to reduce noise. Model parameters are then obtained for concentrations of all anthocyanin species under sample and eluent conditions and are considered together with parameters for anthocyanin species spectral behaviors. The smoothed experimental spectrum then undergoes multiple computational transformations using the quantified parameters, thus generating the final system of deconvoluted spectra for all pigmented anthocyanin species. Lastly, the deconvoluted spectral color characteristics are visualized using colorimetric methods.

The developed deconvolution model is introduced in Figure 2 and outlined below, where the experimental spectrum for a single anthocyanin species gets transformed into a high-fidelity deconvoluted system that represents all existing pigmented species within solution. All simulations for the developed model were conducted using Matlab software (MATLAB R2019b).

#### 2.2.1 Signal Processing

For each sample, the experimentally derived spectrum is denoted here as *h*_exp_(λ), where λ is the wavelength as measured by UV–vis detection. Spectral noise is reduced through the application of a Savitzky–Golay (S–G) filter of the form of eq 1:
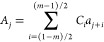
1with parameters defined by eq 2:
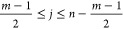
2where spectral data points are represented such that λ_*j*_ is the recorded wavelength and *a*_*j*_ is the recorded absorbance with respect to the degree of association, *j* = 1, 2,..., *n*, . Then *A*_*j*_ is the derivative at point *a*_*j*_, *C*_*i*_ is the set of convolution coefficients, and *m* is the number of convolution coefficients as determined by the order of the smoothing polynomial.^43,44^ Details regarding the convolution coefficients can be found in Supporting Information Section 1. Following S–G filtering, the generated impulse response is the denoised spectrum, called *h*(λ).

#### 2.2.2 Model Parameters

Experimentally validated data for anthocyanin chemical behaviors are introduced to allow for the construction of accurate deconvoluted spectral data. Kinetic and steady state mathematical models (eqs 3–9 and Supporting Information Section 2)^45^ are used to describe the concentration distributions of all monomeric (A^–^, A, AH^+^, B) and self-associated ((A^–^)_*n*_, (A)_*n*_, (AH^+^)_*n*_) anthocyanin species^20^ under sample and eluent conditions. In these models, behaviors for the hemiketal and chalcones are represented together as one species (B), pH is represented by the hydrogen ion concentration [H^+^], and degree of association is denoted as *n*. Kinetic rate constants are denoted as *k*_±_ and *j*_±_ for the monomeric and self-associated species, respectively. The values assigned to *j*_±_ (Supporting Information Section 2) represent anthocyanin self-association kinetics under red wine conditions^46^ (i.e., where self-association is slightly inhibited by winemaking parameters of heat and ethanol)^47^ and in pure solutions where these parameters are absent.^20^ Moreover, the system of kinetic equations (eqs 3–9) can be transformed into steady state equations to represent the system under thermodynamically stable conditions (Supporting Information Section 2), where anthocyanin species distributions at a given time are influenced by parameters of solution pH, flavylium cation concentration [AH^+^], and total anthocyanin concentration [T].
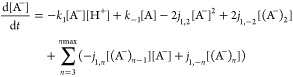
3
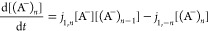
4
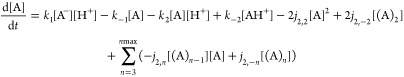
5
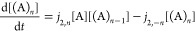
6
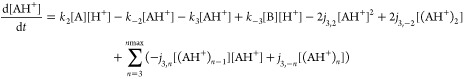
7
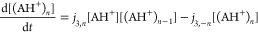
8
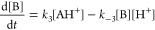
9Using these equations, anthocyanin species profiles are obtained for every sample under conditions corresponding to both the experimental sample and the HPLC-DAD eluent. The model-generated data for all monomeric and self-associated anthocyanin species are then incorporated into Gaussian expressions and used in the numerical FSD system as follows.

From the above simulations, the anthocyanin species are defined within the set:
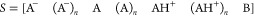
10

Enumerating, the anthocyanin species are represented by
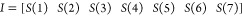
11where each species is indexed by an element *i* ∈ *I*.

Then, the modeled concentration values for the system are described by
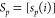
12where (*p* = 1,2) such that *p* = 1 is the pH value of the eluent and *p* = 2 is the pH value of each sample. As such, *S*_1_ corresponds to the concentrations at the eluent pH, *S*_2_ corresponds to the concentrations at the sample pH, and individual anthocyanin species concentrations are represented by the *i*^th^ elements, [*s*_*p*_(*i*)], within both sets.

Next, the relationship between the individual anthocyanin species (obtained experimentally from the spectrum *h*(λ)) and the rest of the species are quantified. According to the Beer–Lambert law, peak absorbance is directly proportional to concentration,^48^ so the absorbance values within the system can be represented as
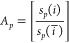
13where *s*_*p*_(*i*) is the concentration of each anthocyanin species *i* and *s*_*p*_(*i̅*) is the concentration of the individual experimentally generated species, for each *s*_*p*_(*i*) ∈ *S*_*p*_. Then the absorbance values of each anthocyanin species (eq 13) are incorporated into spectral expressions to be later transformed with the experimental spectrum *h*(λ) using FSD methods. To describe this, let *G*_*p*_(λ) be two sets containing normal Gaussian distributions:
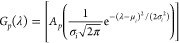
14where *G*_1_(λ) contains the Gaussian distributions at the eluent pH and *G*_2_(λ) contains the Gaussian distributions at the sample pH. Distribution parameters, defined as μ_*i*_ for the mean value that indicates where the peak of density occurs and σ_*i*_ for the standard deviation of the distribution from λ, were calculated for each anthocyanin species *i* within both sets *G*_*p*_(λ).

#### 2.2.3 Spectral Parameters

As introduced in eq 14, the distribution parameters μ_*i*_ and σ_*i*_ are informed experimentally based on the behavior of the collected anthocyanin spectrum, *h*_exp_(λ). In the context of HPLC-DAD analysis, the Gaussian parameter μ is equal to the wavelength of maximum absorbance λ_max_ for a given spectrum. Therefore, the value of μ for the experimental species, denoted as μ_*i̅*_, can be determined experimentally as
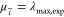
15where λ_max,exp_ is the experimentally measured wavelength of maximum absorbance for the M3G spectrum of each sample. Deprotonation of pigmented anthocyanin species causes bathochromic shifts in the absorbance spectra such that the λ_max_ values for the flavylium cation (AH^+^), quinonoidal base (A), and quinonoidal anion (A^–^) are approximately 520, 570, and 620 nm, respectively.^30^ As such, the experimental species μ_*i̅*_ value can be described as
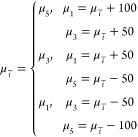
16where μ_5_ is the flavylium cation (AH^+^) parameter, μ_3_ is the quinonoidal base (A) parameter, and μ_1_ is the quinonoidal anion (A^–^) parameter.

Then, as anthocyanin self-association has been shown to cause hypsochromic effects in the visible region of the absorbance spectrum,^31,20^ wavelength values for self-associated species are expressed as functions based on the wavelengths of their corresponding monomeric species and their degrees of self-association. That is, for (AH^+^)_*n*_:
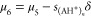
17for (A)_*n*_:
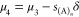
18and for (A^–^)_*n*_:
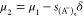
19The constants in these expressions are derived from eqs 3–8 that describe the pigmented species, where *s*_(AH^+^)_*n*__, *s*_(A)_*n*__, and *s*_(A^–^)_*n*__ are the degrees of self-association for each species as determined by the proportion of self-associated and monomeric species (eqs 3–8), and δ is the approximate chemical shift (nm) caused by a single association step, assuming isodesmic self-association.^49,20^ Concentrations for colorless hemiketal and chalcones that contribute to total M3G concentration are predicted by the current study’s models (i.e., as species B, eqs 3–14, and Supporting Information Section 2), but are not displayed in the presented deconvolution outputs as they can absorb maximally at separate wavelengths in the UV region (approximately 280 and 340 nm, respectively) instead of visible ranges (400–700 nm) of the absorbance spectrum.^50^

The experimental species (*i̅*) standard deviation constant σ_*i̅*_ is also determined experimentally from the M3G spectrum of each sample. It is assumed that the proton transfer reactions between anthocyanin species do not generate significant differences in their corresponding spectral bandwidths. Parameter values are therefore assigned according to eq 20:
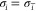
20

#### 2.2.4 Spectral Deconvolution

To perform the multiphase computational deconvolution process, the denoised experimental spectrum for a single species, *h*(λ), is first transformed using modeled values (eqs 3–14) for the anthocyanin species concentrations at the eluent pH, *G*_1_(λ). From this expression, the spectra for all anthocyanin species that would be found in the sample at the eluent pH, *F*_1_(λ) can be obtained:
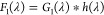
21

Similarly, to generate a representation of anthocyanin behavior under sample pH conditions, each species (*i*) from the expression *F*_1_(λ) is then transformed with experimentally validated values for the same species (*i*) at the sample pH, *G*_2_(λ). This leads to the desired spectra for all anthocyanin species at the sample pH, *F*_2_(λ), written as
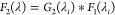
22Further numerical analysis of the spectral deconvolution process is provided in Supporting Information Section 3.

#### 2.2.5 Colorimetry

For each sample, the experimental spectrum for a single anthocyanin species (Sections 2.1 and 2.2.1) and final system of deconvoluted spectra for all species (Section 2.2.4) are represented as RGB coordinates using the colorimetric method presented previously.^46^ Resultant color profiles are represented visually using ColorHexa software,^51^ with additional details and visualized outputs provided in Supporting Information Sections 5 and 6.

## 3 Results and Discussion

### 3.1 Spectral Deconvolution under Acidic Conditions

The developed multiphase deconvolution model is outlined in Figure 2 and provided in its entirety within Section 2.2 and Supporting Information Sections 1–3. The model can be used to transform experimental spectral data for anthocyanins within acidic solutions (0.0 < pH < 4.0) such as red wines, plant extracts, dyes, and pH-sensors.^28,8,52,29^ A case study demonstrating the model is presented below (Section 3.1.1) that transforms spectral data of model anthocyanin, M3G, from a commercial Shiraz fermentation (W_1_), with additional establishment of the model’s application for red wine analysis provided in Supporting Information Section 4.

#### 3.1.1 Model Initiation, Parameter Derivations, and Spectral Transformations

**Figure 3:**
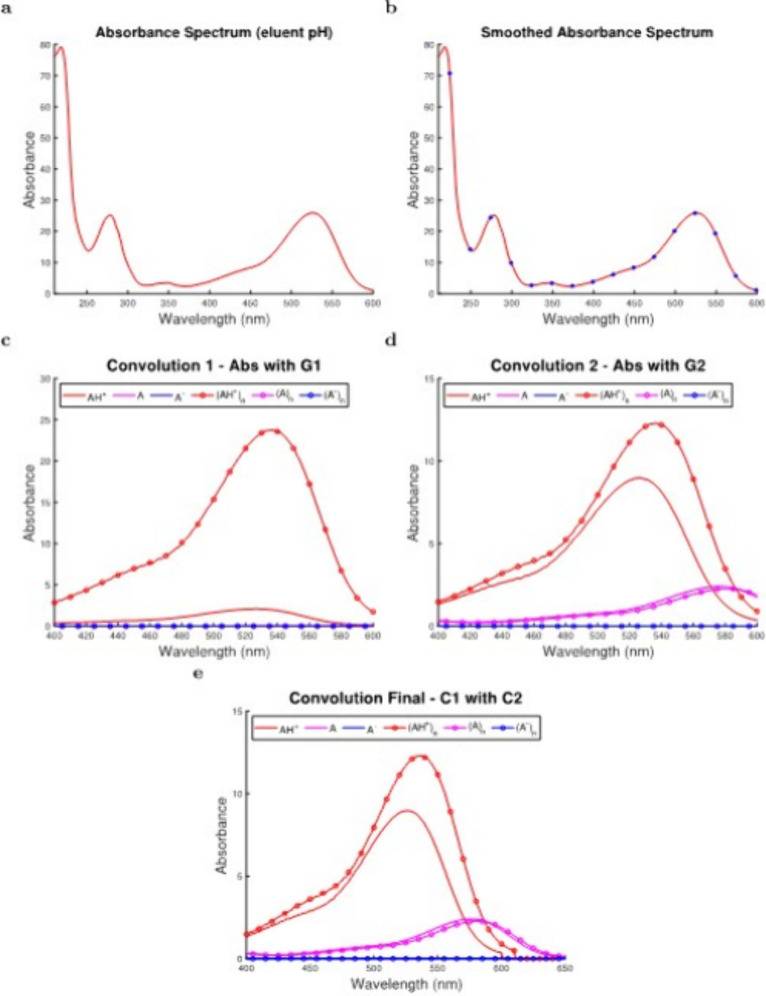
A schematic workflow of the developed deconvolution process, as illustrated using data from the first collected sample (SI) from W_1_. Stages of the deconvolution method are represented by the experimentally collected spectrum, showing (a) the flavylium cation (AH^+^) form, (b) the spectrum during the smoothing process, (c) the first convolution of the experimental spectrum (C1) with Gaussian parameters (*G*_1_) corresponding to the eluent pH, (d) the second convolution of the experimental spectrum (C2) with Gaussian parameters (*G*_2_) corresponding to the wine sample pH, (e) and the convolution of C1 and C2, resulting in the final deconvoluted (CF) spectra of all pigmented anthocyanin species that exist in solution.

Initiating the developed deconvolution model (Section 2.2), the experimental spectrum for initial fermentation sample (SI) of W_1_ was imported into Matlab software (MATLAB R2019b) (Figure 3a). A Savitzky–Golay smoothing filter was then used to reduce spectral noise acquired during laboratory sample analysis (Figure 3b and eqs 1 and 2).

Experimentally validated parameters were obtained by inputting measured values (Section 2.1) for total M3G concentration (2.34 × 10^–5^ M), wine sample pH (3.43), and eluent pH (2.50) into the employed system of kinetic equations for all anthocyanin species (eqs 3–9). Values for equilibrium (*J*) and kinetic rate constants (*j*_±_) were chosen to reflect anthocyanin self-association behaviors under red wine conditions (Supporting Information Section 2).^46^ From here, concentrations of all monomeric and self-associated anthocyanin species were obtained under conditions corresponding to the eluent (Table 1, condition a) and wine sample (Table 1, condition c), where the flavylium cation and quinonoidal base were the dominating pigmented species due to solution pH^17^ and hemiketal formation was inhibited by self-association of pigmented monomers (eqs 3–9 and Supporting Information Section 2).^20,46,16^ Ratios of each species with respect to the monomeric flavylium cation AH^+^ (e.g., A^–^/AH^+^) were then obtained for both systems, generating Gaussian parameters (eqs 10–14) for the eluent (*G*_1_) and wine sample (*G*_2_) conditions (Table 1, conditions b and d). Lastly, behaviors of the pigmented anthocyanin species were identified (Table 1, conditions Pa–Pd) for implementation into the spectral deconvolution model, which were shown by the model (eqs 3–9 and Supporting Information Section 2) to have a summed M3G concentration of 2.32 × 10^–5^ M at the eluent pH (Table 1, condition Pa), 1.98 × 10^–5^ M at the wine sample pH (Table 1, condition Pc), and the same ratios of each species with respect to AH^+^ (Table 1, conditions Pb and Pd) as in the system with the hemiketal (Table 1, conditions b and d).

**Table 1:** Modelled Values Represent (Condition a) the Concentrations (M) of All Monomeric (A^–^, A, AH^+^, B) and Self-Associated ((A^–^)_*n*_, (A)_*n*_, (AH^+^)_*n*_) Anthocyanin Species at the Eluent pH, (Condition b) Gaussian Eluent Concentration Ratios (*G*_1_) of All Species with Respect to the Monomeric Flavylium Cation AH^+^(e.g., A^–^/AH^+^), (Condition c) Modelled Concentrations of All Species at the Wine Sample pH, and (condition d) Gaussian Wine Sample Concentration Ratios (*G*_2_) with respect to AH^+^^a^

cond.	A^–^	A	AH^+^	B	(A^–^)_*n*_	(A)_*n*_	(AH^+^)_*n*_	total M3G
a	2.95 × 10^–12^	5.89 × 10^–8^	1.86 × 10^–6^	1.13 × 10^–7^	1.90 × 10^–18^	9.48 × 10^–9^	2.13 × 10^–5^	2.34 × 10^–5^
b	1.58 × 10^–6^	3.16 × 10^–2^	1	6.08 × 10^–2^	1.02 × 10^–12^	5.09 × 10^–3^	11.45	
c	7.91 × 10^–10^	1.84 × 10^–6^	6.87 × 10^–6^	3.54 × 10^–6^	1.74 × 10^–14^	1.74 × 10^–6^	9.38 × 10^–6^	2.34 × 10^–5^
d	1.15 × 10^–4^	0.27	1	0.52	2.53 × 10^–9^	0.25	1.37	

cond.	A^–^	A	AH^+^	B	(A^–^)_*n*_	(A)_*n*_	(AH^+^)_*n*_	pig. M3G
Pa	2.95 × 10^–12^	5.89 × 10^–8^	1.86 × 10^–6^	0	1.90 × 10^–18^	9.48 × 10^–9^	2.13 × 10^–5^	2.32 × 10^–5^
Pb	1.58 × 10^–6^	3.16 × 10^–2^	1	0	1.02 × 10^–12^	5.09 × 10^–3^	11.45	
*Pc*	7.91 × 10^–10^	1.84 × 10^–6^	6.87 × 10^–6^	0	1.74 × 10^–14^	1.74 × 10^–6^	9.38 × 10^–6^	1.98 × 10^–5^
Pd	1.15 × 10^–4^	0.27	1	0	2.53 × 10^–9^	0.25	1.37	

a For each condition (a–d), the table highlights (conditions Pa–Pd) values for the pigmented species (A^–^, A, AH^+^, (A^–^)_*n*_, (A)_*n*_, (AH^+^)_*n*_) that get incorporated into the deconvolution model.

Spectral transformations could then occur, whereby modeled parameters for pigmented anthocyanin species (Table 1 and eqs 1–14) were incorporated with corresponding experimental spectral parameters for each species (eqs 15–20) into multiphase Fourier spectral deconvolution equations (eqs 21 and 22 and Supporting Information Section 3). The experimental spectrum (Figure 3a) was separately convolved with *G*_1_, corresponding with the eluent pH (Figure 3c), and *G*_2_, corresponding with the wine sample pH (Figure 3d). Finally, each of the convoluted spectra underwent convolution with each other, which generated the final system of deconvoluted spectra for all pigmented anthocyanin species in SI (Figure 3e).

#### 3.1.2 Insights from High-Fidelity Deconvoluted Spectra

HPLC-DAD signal acquisition (Figure 3a) and smoothing (Figure 3b) indicated the experimental spectrum exhibited a maximum absorbance at approximately 520 nm and was comprised entirely of the red monomeric flavylium cation, due to acidic conditions that were required for analysis (Figure 3a,b).^53^ Results were considered for intermediate convolutions of pigmented anthocyanin species that occurred at eluent (C1) (Figure 3c) and wine sample (C2) (Figure 3d) pH conditions to increase fidelity of the experimental data using the developed deconvolution model (Section 2.2 and Supporting Information Sections 1–3). When the sample was under analytical (e.g., eluent) conditions, high levels of acidity (pH = 2.50) resulted in the monomeric and self-associated flavylium cation being the only majorly present species (Figure 3c and eqs 3–9), in accordance with previous findings.^16,17^ A large amount of the dark red self-associated flavylium cation was observed in particular (2.13 × 10^–5^ M, Table 1, condition Pa), as self-association of AH^+^ was not inhibited by other species in the reaction network at this pH^46,14,15^ (Supporting Information Section 2).

In comparison, when the sample was under its actualised pH conditions (Table 1 and Figure 3d), modeled data revealed that the purple monomeric and dark purple self-associated quinonoidal base species were present in larger quantities (i.e., 1.84 × 10^–6^ M of A and 1.74 × 10^–6^ M of (A)_*n*_, Table 1, condition *Pc*) due to the sample possessing a pH of 3.43 (eqs 3–9 and Supporting Information Section 2), again demonstrating the high-fidelity capabilities of the developed work to identify pH-dependent species (Figure 3c). Decreased self-association of (AH^+^)_*n*_ and a higher concentration of total pigmented monomers (i.e., A^–^ + A + AH^+^) were also observed (8.71 × 10^–6^ M, Table 1, condition *Pc*, and Figure 3d) compared to what existed in the eluent system (1.92 × 10^–6^ M, Table 1, condition Pa, and Figure 3c) as expected, due to competition of the quinonoidal base and flavylium cation species within the multistate system caused by sample pH.^19,20^ Additionally, hypsochromic and bathochromic effects caused deconvoluted spectra for (AH^+^)_*n*_, A, and (A)_*n*_ to absorb at higher wavelengths (i.e., approximately 530, 570, and 580 nm, respectively, Figure 3e) compared to the experimental flavylium-form spectrum that absorbed at approximately 520 nm (Figure 3a).^30^ Both intermediate convolutions simulated by the model (Figure 3c,d) provided more accurate data for characterizing anthocyanins and increased levels of signal feature extraction compared to what was provided by HPLC-DAD data (Figure 3a).

Systems for C1 (Figure 3c) and C2 (Figure 3d) were then convolved together (Figure 3e) to incorporate the modeled wine sample parameters (Figure 3d) with spectral characteristics (i.e., periodicity) that were obtained experimentally (Figure 3a) and preserved in the data for analytical (i.e., eluent) systems (Figure 3b,c). This allowed for spectral data to be accurately deconvoluted across chosen regions of Vis spectra (e.g., 400–650 nm for SI, Figure 3e), including regions where experimental data were not collected (e.g., 600–650 nm for SI, Figure 3a). As a result, the experimental spectrum that represented a single anthocyanin species (i.e., AH^+^) under analytical conditions (Figure 3a) was transformed into a spectral system that revealed behaviors displayed by each pigmented anthocyanin species (i.e., A^–^, A, AH^+^, (A^–^)_*n*_, (A)_*n*_, (AH^+^)_*n*_) in actuality within the analyzed sample (Figure 3e). Representing this phenomenon, colorimetric methods and resultant color profiles are provided for experimental and deconvoluted spectra of W_1_ (Figure 3a,e) and other commercial fermentation samples (Supporting Information Section 5).

It is also noted that the summed concentration of pigmented species in the sample (1.98 × 10^–5^ M) was found to be slightly lower (and perhaps a more useful indicator for predicting and controlling sample color) than the measured value for total M3G concentration obtained by HPLC methods (2.34 × 10^–5^ M, Table 1), which included the concentration of the colorless hemiketal species that was presented under the HPLC conditions as M3G in flavylium form. By providing data for all pigmented monomeric and self-associated anthocyanin species, the deconvoluted system (Figure 3e) generated powerful information about sample color and stability characteristics that were not available from HPLC data for total M3G concentration on its own (Figure 3a).

### 3.2 Spectral Deconvolution under Neutral and Basic Conditions

While anthocyanin-containing products are typically produced and analyzed under acidic conditions to facilitate anthocyanin detection and stabilization^28,1,29^ (Section 3.1), there are several instances where the purple quinonoidal base (A) and blue quinonoidal anion (A^–^) are the primarily investigated species.^54,7,9,1^ Addressing this prospect, the present study’s deconvolution model (Section 2.2 and Supporting Information Sections 1–3) has been used to transform hypothetical experimental spectra under neutral (4.0 < pH < 7.0) and basic (7.0 < pH < 9.0) conditions to reflect behaviors exhibited by all pigmented anthocyanin species in solution.

**Figure 4:**
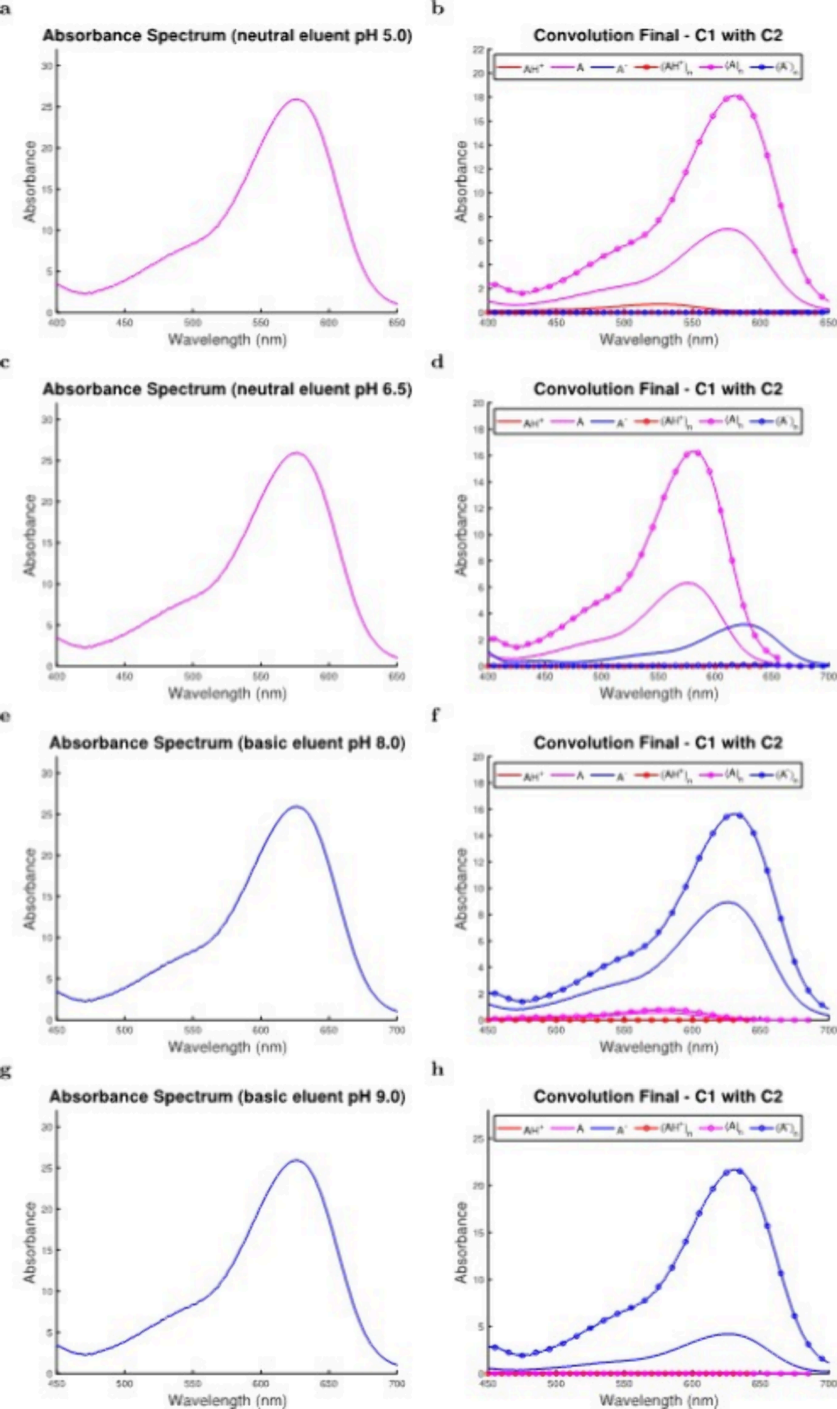
Hypothetical spectra are simulated for solutions possessing a total M3G concentration of 2.34 × 10^–5^ M and pH values of (a) 5.0, (c) 6.5, (e) 8.0, and (g) 9.0. The spectra are deconvoluted using model eluent pH values that are consistent with the pH values chosen for the samples, and resultant deconvoluted systems for all pigmented anthocyanin species are provided in (b), (d), (f), and (h), respectively.

Hypothetical absorbance spectra were generated for samples possessing total M3G concentrations of 2.34 × 10^–5^ M (which is the recorded value from SI of W_1_) (Table 1 and Figure 3a), and pH values of the sample and eluent were assigned to be equal under neutral (i.e., pH = 5.0, 6.5) and basic (i.e., pH = 8.0, 9.0) conditions (Figure 4a,c,e,g). Hypothetical spectra were comprised of the purple quinonoidal base (A) in neutral samples (Figure 4a,c) and blue quinonoidal anion (A^–^) in basic samples (Figure 4e,g), owing to the pH-dependence of the anthocyanin multistate system (eqs 3–9 and Supporting Information Section 2).^16,17^ Values of λ_max_ were approximately 570 nm for A (Figure 4a,c) and 620 nm for A^–^ (Figure 4e,g), due to bathochromic shifts caused by deprotonation of pigmented anthocyanin species (e.g., from AH^+^ that exhibited a maximum absorbance at approximately 520 nm) (Figure 3a).^30^ Gaussian parameters were constructed (eqs 1–14) for each hypothetical absorbance spectrum (Figure 4a,c,e,g and Supporting Information Section 6) to implement the developed deconvolution model, and self-association rate constants were chosen to represent associative kinetics in pure solutions (Supporting Information Section 2),^18,20^ where interactions are not inhibited by heat and ethanol.^47^

Deconvoluted data (Figure 4b,d,f,h) showed that self-associated species would be more prevalent than monomers in all hypothetical samples (Figure 4a,c,e,g) since anthocyanins exhibit nonlinear self-association kinetics at this M3G concentration (2.34 × 10^–5^ M, Supporting Information Section 2) as demonstrated in previous findings,^46^ with hypsochromic effects causing λ_max_ values to be approximately 580 nm for (A)_*n*_ (Figure 4b,d) and 630 nm for (A^–^)_*n*_ (Figure 4f,h).^31,20^ Hypothetical samples also reflected the pH-dependent nature of the reaction network (eqs 3–9 and Supporting Information Section 2)^16,17^ that was observed under acidic conditions (Section 3.1), as the deconvoluted systems gradually shifted from being dominated by the quinonoidal base species (Figure 4b) to the quinonoidal anion species (Figure 4h) as pH increased (i.e., from 5.0 in Figure 4b to 9.0 in Figure 4h). Corresponding colorimetric profiles and concentrations of self-associated species (i.e., indicating temporal stability) for hypothetical experimental (Figure 4a,c,e,g) and deconvoluted (Figure 4b,d,f,h) spectra are provided in Supporting Information Section 6. In all instances (Figure 4 and Supporting Information Section 6), deconvoluted data were shown to possess significantly increased complexity compared to simulated experimental data with chosen conditions of total M3G concentration and pH. As a result, the developed computational approach (eqs 1–22 and Supporting Information Sections 1–3) has potential to accurately represent behaviors of A and A^–^, while also limiting their exposure to experimental conditions that could destabilize them (e.g., through hemiketal formation).^29,1,55^

### 3.3 Future Directions

Moving forward, the present approach could be modified (i.e., by adjusting the reactions constants employed in eqs 3–9 and Supporting Information Section 2)^46^ to describe anthocyanins besides M3G that can display blue-red (delphinidin, petunidin), pink-red (peonidin), orange-red (cyanidin), and orange (pelargonidin) hues in plant organs (e.g., fruits, flowers, leaves) and red wines.^2−5,21,26^ Anthocyanin-related compounds existing in comparable multistate systems (e.g., furanoflavylium cations, 3-deoxyanthocyanidins)^56,57^ and acylated anthocyanins that can exhibit varied color expression and stabilization mechanisms compared to nonacylated forms^58,59^ could also be accommodated.

Capabilities of the present work to identify small amounts of pigmented species, as demonstrated in the deconvoluted system when pH = 5.0 by small amounts of AH^+^ being present (i.e., AH^+^ concentration of 4.14 × 10^–7^ M compared to the total M3G concentration of 2.34 × 10^–5^ M, Section 3.2, and Figure 4b), could also be utilized. Potential applications include assisting with the interpretation of results from pH-sensors (0.0 < pH < 9.0), informing product stability and shelf life, and predicting formation of compounds including metal or intermolecular copigment complexes, tannin/anthocyanin adducts, and pyranoanthocyanins that arise from reactions with pigmented monomers.^7−9,21,23,60,61^ Moreover, additional reactions that occur under highly alkaline conditions (pH > 9.0) (e.g., that generate B^–^, B^2–^, and increase chalcone formation) could be accommodated by the developed model (Section 2.2 and Supporting Information Sections 1–3) in the future.^20,62−64^

In total, Fourier spectral deconvolution methods were used to transform experimental spectra for pigmented anthocyanin monomers into systems that represent spectral behaviors of all pigmented monomeric and self-associated anthocyainin species in solution. The developed computational model was found to significantly increase the level of signal feature extraction for spectral data of anthocyanins, and thereby provided key information about anthocyanin color expression and stability characteristics that were not attainable using traditional HPLC with UV–vis detector methods alone. The ability of the present study to enhance the fidelity and accuracy of anthocyanin spectra could potentially have broad applications for the analysis and production of anthocyanin-containing dyes, textiles, cosmetics, pH-sensors, foods, beverages, and red wines.

## Data Availability

All experimental and simulated data are reported in the article and Supporting Information.

## Supporting Information Available

Parameters for Savitzky–Golay convolution coefficients; equilibrium and rate constants for all pigmented monomeric and self-associated anthocyanin species; steady state equations for all anthocyanin species; derivation of mathematical deconvolution equations; signal filtering equations; additional establishment of the developed deconvolution model for analysis of commercial red wines; details for a computational colorimetry method to describe all pigmented monomeric and self-associated anthocyanin species; colorimetric profiles for the experimental and deconvoluted data of supplemental red wine samples; modeled Gaussian parameters for all pigmented species under neutral and basic conditions; colorimetric profiles for presented experimental and deconvoluted data under neutral and basic conditions (PDF)

## Abbreviations

HPLC-DAD: High-performance liquid chromatography with diode array detection
AH^+^: flavylium cation
A: quinonoidal base
A^–^: quinonoidal anion
B: hemiketal
*Cc*: *cis*-chalcone
Ct: *trans*-chalcone
(AH^+^)_*n*_: self-associated flavylium cation
(A)_*n*_: self-associated quinonoidal base
(A^–^)_*n*_: self-associated quinonoidal anion
FSD: Fourier spectral deconvolution
M3G: malvidin-3-*O*-β-d-glucopyranoside
S–G: Savitzky–Golay
