# Supplementary material for: Fourier Spectral Deconvolution to Describe Behaviors of pH-Dependent Monomeric and Self-Associated Anthocyanin Species
Source: J Chem Inf Model. 2025 Mar 3;65(6):2834–44. doi: 10.1021/acs.jcim.4c02300 (PMC11939003; doi:10.1021/acs.jcim.4c02300)
Supplement: Supplementary file 1 — ci4c02300_si_001.pdf [file ci4c02300_si_001.pdf]

Fourier spectral deconvolution to describe behaviours of pH-dependent monomeric and self-associated anthocyanin species

Rachael A. Tindal [a], David W. Jeffery [a], Richard A. Muhlack [a,*]

[a] Australian Research Council Training Centre for Innovative Wine Production and Waite Research Institute, The University of Adelaide, PMB 1, Glen Osmond, South Australia 5064, Australia

* Corresponding author.

E-mail address: richard.muhlack@adelaide.edu.au (R. A. Muhlack).

Table of Contents:

1. Savitzky-Golay Convolution Coefficients 3
2. Monomeric and Self-Associated Anthocyanin Species Model Parameters......3
  2.1. Rate Constants 3
  2.2. Steady State Equations 4
3. Deconvolution Process 4
  3.1. Deconvolution Derivations 4
  3.2. Signal Filtering 6
4. Supplemental Red Wine Case Studies (W_1 Throughout Fermentation)......6
  4.1. W_1 Throughout Fermentation 6
  4.2. Commercial Fermentations W_2 and W_3......9
5. Colourimetry......13
  5.1. Colourimetry Method 13
  5.2. Colourimetric Profiles for W_1......14
  5.3. Colourimetry with Concentration-Dependent Coordinates 15
6. Spectral Deconvolution in Neutral and Basic Simulations......16
  6.1. Modelled Gaussian Parameters for all Pigmented Species 16
  6.2. Colourimetry for Neutral and Basic Spectra 17

1. Savitzky-Golay Convolution Coefficients

The experimental spectrum $h_{exp}(\lambda)$ must be continuous and smooth across all data points $(x_i, y_i) \in h_{exp}(\lambda), i \in \mathbb{Z}^+$. That is, for all $x_i \in h_{exp}(\lambda)$, there exists a corresponding real value $y_i \in h_{exp}(\lambda)$. Furthermore, the distance between each abscissa (x–coordinate) value is equal such that $(x_i - x_{i-1}) = (x_{i-1} - x_{i-2})$ for all $x_i \in h_{exp}(\lambda)$.

2. Monomeric and Self-Associated Anthocyanin Species Model Parameters

2.1. Rate Constants

Equilibrium and rate constants for monomeric and self-associated anthocyanin species

Equilibrium (K, J) or rate ($k_{\pm}, j_{\pm}$) constant	Value	Source
K_1 (M)	$10^{-6.8}$	5
k_1 ($M^{-1}s^{-1}$)	1	
k_{-1} (s^{-1})	$10^{-6.8}$	
K_2 (M)	10^{-4}	6
k_2 ($M^{-1}s^{-1}$)	1	
k_{-2} (s^{-1})	10^{-4}	
K_3 (M)	$10^{-1.92}$	5, 7
k_3 ($M^{-1}s^{-1}$)	100	
k_{-3} (s^{-1})	1.2	
J_1 (M^{-1})	$< J_3$	8
J_2 (M^{-1})	$> J_3$	
J_3 (M^{-1})	$10^2 < J_3 < 10^4$	
j_i ($M^{-1}s^{-1}$)	$10^1 - 10^{2.5}$	
j_{-i} (s^{-1})	$10^{-3} - 10^{-1.5}$	
J_1 (M^{-1})	$10^{5.25}$	2
J_2 (M^{-1})	$10^{6.4}$	
J_3 (M^{-1})	$10^{6.05}$	
j_1 ($M^{-1}s^{-1}$)	$10^{2.25}$	
j_2 ($M^{-1}s^{-1}$)	$10^{2.4}$	
j_3 ($M^{-1}s^{-1}$)	$10^{2.45}$	
J_1 (M^{-1})	$10^{5.15}$	3
J_2 (M^{-1})	$10^{6.2}$	
J_3 (M^{-1})	$10^{5.715}$	
j_1 ($M^{-1}s^{-1}$)	$10^{2.15}$	
j_2 ($M^{-1}s^{-1}$)	$10^{2.2}$	
j_3 ($M^{-1}s^{-1}$)	$10^{2.115}$	
j_{-1} (s^{-1})	10^{-3}	2, 3
j_{-2} (s^{-1})	10^{-4}	
j_{-3} (s^{-1})	$10^{-3.6}$	

Table S1. Equilibrium constants for monomeric (K) and self-associated (J) anthocyanins, with rate constants ($k_{\pm}, j_{\pm}$) derived from these values such that $K_i = \frac{k_i}{k_{-i}}$ and $J_i = \frac{j_i}{j_{-i}}$. Rate constants represent anthocyanin system behaviours[1] in pure solutions[2] and under red wine conditions[3], where self-associative behaviour is slightly inhibited by winemaking parameters of heat and ethanol[4].

As mentioned in the main text (Section 3.3), the present work could be modified by adjusting the reactions constants shown in Table S1, employed in Equations 3–9 of the main text and Equations S.1–S.7), to describe other anthocyanins besides M3G that can display various hues. These include compounds that provide blue-red (delphinidin, petunidin), pink-red (peonidin), orange-red (cyanidin), and orange (pelargonidin) colours[9,10,11,12]. Modification of the employed reaction constants (Table S1) could also allow for the present work to describe anthocyanin-related compounds that exist in comparable multistate systems, including furanoflavylium cations such as riccionidin A, whose species display yellow, pink and purple hues, and 3–deoxyanthocyanidins such as luteolinidin, whose species display orange and red hues[13,14]. The present study may therefore have opportunities for applications across various research and industry sectors to help facilitate chemical analysis, stabilisation, and commercialisation of anthocyanins.

2.2. Steady State Equations

Steady state equations derived from the kinetic system of equations (eq. 3–9 of the main text) for monomeric and self-associated anthocyanin species are provided here. Equations are guided by the monomeric flavylium cation AH$^+$and are written with respect to the total M3G concentration in the system [T].

$$[\mathrm{B}] = K_3 \frac{1}{[\mathrm{H}^+]} \mathrm{AH}^+ (S1)$$

$$[\mathrm{AH}^+] = \frac{[\mathrm{T}]}{K_1 K_2 \frac{1}{[\mathrm{H}^+]^2} + K_2 \frac{1}{[\mathrm{H}^+]} + 1 + K_3 \frac{1}{[\mathrm{H}^+]} + \left(nJ_1 \left(K_1 K_2 \frac{1}{[\mathrm{H}^+]^2} \right)^n + nJ_2 \left(K_2 \frac{1}{[\mathrm{H}^+]} \right)^n + nJ_3 \right)^{\frac{1}{n}}}$$

$$[(\mathrm{AH}^+)_\mathrm{n}] = J_3 [\mathrm{AH}^+]^n (S3)$$

$$[\mathrm{A}] = K_2 \frac{1}{[\mathrm{H}^+]} [\mathrm{AH}^+] (\mathrm{S4})$$

$$[(\mathrm{A})_\mathrm{n}] = J_2 \left(K_2 \frac{1}{[\mathrm{H}^+]} [\mathrm{AH}^+] \right)^n (\mathrm{S5})$$

$$[\mathrm{A}^-] = K_1 K_2 \frac{1}{[\mathrm{H}^+]^2} [\mathrm{AH}^+] (\mathrm{S6})$$

$$[(\mathrm{A}^-)_\mathrm{n}] = J_1 \left(K_1 K_2 \frac{1}{[\mathrm{H}^+]^2} [\mathrm{AH}^+] \right)^n (S7)$$

3. Deconvolution Process

3.1. Deconvolution Derivations

First, the convolution incorporating parameters from the eluent pH (eq. 21 of the main text) can be represented as:

$$F_1(\lambda) = G_1(\lambda) * h(\lambda) (S8)$$

where for each sample, $G_1(\lambda)$ is a vector of Gaussian functions:

$$G_1(\lambda) = \frac{1}{\sigma\sqrt{2\pi}} e^{-\frac{(\lambda-\mu)^2}{2\sigma^2}} (S9)$$

where the mean $\mu = [\mu_1 \quad \mu_2 \quad \cdots \quad \mu_7]$ and standard deviation $\sigma = [\sigma_1 \quad \sigma_2 \quad \cdots \quad \sigma_7]$ are values corresponding to the seven anthocyanin species, $S = [\mathrm{A}^- \quad (\mathrm{A}^-)_\mathrm{n} \quad \mathrm{A} \quad (\mathrm{A})_\mathrm{n} \quad \mathrm{AH}^+ \quad (\mathrm{AH}^+)_\mathrm{n} \quad \mathrm{B}]$, at the

eluent pH. Further, $h(\lambda)$ is the experimental M3G spectrum, and F_1, G_1 and h are functions of wavelength (nm), λ. Then, the Fourier transform of the convolution can be described as:

$$\mathcal{F}_1(G_1(\lambda) * h(\lambda)) = \int_{-\infty}^{\infty} G_1(\tau) \cdot h(\lambda - \tau) d\tau (S10)$$

Letting $(\lambda - \tau) = \omega$, this can be rewritten by definition as:

$$\mathcal{F}_1(G_1(\lambda) * h(\lambda)) = \int_{-\infty}^{\infty} G_1(\tau) \left[\int_{-\infty}^{\infty} \mathcal{H}(\omega) e^{-\beta\omega(\lambda-\tau)} d\omega \right] d\tau (S11)$$

and swapping the order of integration gives:

$$\mathcal{F}_1(G_1(\lambda) * h(\lambda)) = \int_{-\infty}^{\infty} \mathcal{H}(\omega) \left[\int_{-\infty}^{\infty} G_1(\tau) e^{\beta\omega\tau} d\tau \right] e^{-\beta\omega\lambda} d\omega (S12)$$

This is equal to the sum of the Fourier transforms for $G_1(\lambda)$ and $h(\lambda)$, where the Fourier transforms are expressed as:

$$\mathcal{G}_1[G_1](\omega) = \mathcal{G}_1[g_1](\omega) = e^{2\pi\beta\omega\mu} e^{-2\pi^2\omega^2\sigma^2} (S13)$$

and

$$\mathcal{H}[h](\omega) = \int_{-\infty}^{\infty} h(\lambda) e^{-\beta\omega\lambda} d\lambda (S14)$$

Summing the Fourier transforms for $G_1(\lambda)$ and $h(\lambda)$ gives:

$$\mathcal{F}_1(G_1(\lambda) * h(\lambda)) = [\mathcal{G}_1(\omega)\mathcal{H}(\omega)] (S15)$$

which is equal to:

$$e^{2\pi\beta\omega\mu} e^{-2\pi^2\omega^2\sigma^2} \int_{-\infty}^{\infty} h(\lambda) e^{-\beta\omega\lambda} d\lambda (S16)$$

Then, taking the inverse Fourier transform of this expression gives:

$$F_1(\lambda) = [\mathcal{F}_1(G_1(\lambda) * h(\lambda))]^{-1} (S17)$$

which is equal to:

$$\frac{1}{2\pi} \int_{-\infty}^{\infty} (\mathcal{F}_1(G_1(\lambda) * h(\lambda))) e^{\beta\omega\lambda} d\omega (S18)$$

The deconvolved spectra of all anthocyanin species at the eluent pH can therefore be represented by the vector:

$$F_1(\lambda) = \frac{1}{2\pi} \int_{-\infty}^{\infty} \left[e^{2\pi\beta\omega\mu} e^{-2\pi^2\omega^2\sigma^2} \int_{-\infty}^{\infty} h(\lambda) e^{-\beta\omega\lambda} d\lambda \right] e^{\beta\omega\lambda} (S19)$$

Continuing this numerical process, spectra of all anthocyanin species can be obtained for each sample at conditions corresponding to the wine sample pH values. A vector of Gaussian functions is denoted $G_2(\lambda)$, where:

$$G_2(\lambda) = \frac{1}{\sigma'\sqrt{2\pi}} e^{-\frac{(\lambda-\mu')^2}{2\sigma'^2}} (S20)$$

such that μ'_i, σ'_i are the ith elements of $\mu'_i = [\mu'_1 \quad \mu'_2 \quad \cdots \quad \mu'_7]$, $\sigma'_i = [\sigma'_1 \quad \sigma'_2 \quad \cdots \quad \sigma'_7]$, which are the mean and standard deviation values corresponding to the anthocyanin species $S = [\mathrm{A}^- \quad (\mathrm{A}^-)_\mathrm{n} \quad \mathrm{A} \quad (\mathrm{A})_\mathrm{n} \quad \mathrm{AH}^+ \quad (\mathrm{AH}^+)_\mathrm{n} \quad \mathrm{B}]$, at the wine sample pH.

Then, each anthocyanin species can be described as an element $s_i \in S = [\mathrm{A}^- \quad (\mathrm{A}^-)_\mathrm{n} \quad \mathrm{A} \quad (\mathrm{A})_\mathrm{n} \quad \mathrm{AH}^+ \quad (\mathrm{AH}^+)_\mathrm{n} \quad \mathrm{B}]$, where i = 1, 2, ..., 7. As such, the behaviour of each anthocyanin species is represented by the ith entries of vectors $G_2(\lambda)$ and $F_1(\lambda)$, and the convolution incorporating parameters from the wine sample pH (eq. 22 of the main text) is:

$$F_2(\lambda) = G_2(\lambda_i) * F_1(\lambda_i), (S21)$$

which is equal to:

$$\int_{-\infty}^{\infty} G_2(\tau_i) \cdot F_1(\lambda_i - \tau_i) d\tau_i (S22)$$

for all i. This can be reduced to give:

$$\mathcal{F}_2(G_2(\lambda_i) * F_1(\lambda_i)) = \int_{-\infty}^{\infty} \mathcal{F}_1(\omega_i) \left[\int_{-\infty}^{\infty} G_2(\tau_i) e^{\beta_i \omega_i \tau_i} \, d\tau_i \right] e^{-\beta_i \omega_i \lambda_i} d\omega_i (S23)$$

with the Fourier transforms of $G_2(\lambda_i)$ and $F_1(\lambda_i)$ being:

$$\mathcal{G}_2[G_2](\omega_i) = \mathcal{G}_2[g_2](\omega_i) = e^{2\pi\beta_i\omega_i\mu_i'} e^{-2\pi^2\omega_i^2\sigma'^2} (S24)$$

and

$$\mathcal{F}_1[F_1](\omega_i) = \int_{-\infty}^{\infty} F_1(\lambda_i) \, e^{-\beta_i \omega_i \lambda_i} d\lambda_i (S25)$$

for all i. Then:

$$\mathcal{F}_2(G_2(\lambda_i) * F_1(\lambda_i)) = [\mathcal{G}_2(\omega_i) \mathcal{F}_1(\omega_i)] (S26)$$

and the expression for the spectra of all anthocyanin species at the wine sample pH is therefore equal to the vector:

$$F_2(\lambda) = [\mathcal{G}_2(\omega_i) \mathcal{F}_1(\omega_i)]^{-1} (S27)$$

which can be simplified for all i as:

$$\frac{1}{2\pi} \int_{-\infty}^{\infty} \left[e^{2\pi\beta_i\omega_i\mu_i'} e^{-2\pi^2\omega_i^2\sigma'^2} F_1(\lambda_i) e^{-\beta_i\omega_i\lambda_i} d\lambda_i \right] e^{\beta_i\omega_i\lambda_i} d\omega_i (S28)$$

3.2. Signal Filtering

To conclude the convolution process, all signals in $F_2(\lambda)$ are passed through an ideal low pass filter (l) to minimise any existing peak tailing and low-frequency noise that was present in the experimental spectra. By taking the wavelength range of each spectrum, a size-appropriate ideal filter can be constructed of the form:

$$l(\lambda) = \begin{cases} 1, & u_{min} \leq \lambda \leq u_{max} \\ 0, & \text{otherwise} \end{cases} (S29)$$

where u_{min} and u_{max} are the minimum and maximum wavelength values for all functions $F_2(\lambda_i) \in F_2$ (λ). Then:

$$F_2(\lambda) = F_2(\lambda) * l(\lambda) (S30)$$

4. Supplemental Red Wine Case Studies

4.1. W_1 Throughout Fermentation

Providing additional establishment of the developed deconvolution model (Section 2.2 of the main text and Supporting Information sections 1–3) to be applied for red wine analysis, additional samples of commercial fermentation W_1 (Sections 2.1 and 3.1 of the main text) collected at various stages (i.e., first half (H1), second half (H2) and final (F)) of the fermentation process were considered. Recorded HPLC data for total M3G concentration and empirical data for pH of each sample (Section 2.1 of the main text and Table S2) were used to generate model-derived parameters for the eluent and wine sample pH systems (eq. 3–9 of the main text and Table S3) of each sample, with values for equilibrium

(J) and kinetic rate ($j_{\pm}$) constants chosen to reflect anthocyanin self-association behaviours under red wine conditions (Supporting Information section 2)[3]. Using the modelled data (Table S3), the developed deconvolution methods (Section 2.2 of the main text and Supporting Information section 3) were applied to the HPLC-DAD spectra of all samples (Figure S1).

Winery data for W_1 throughout fermentation

S	Total M3G Concentration (M)	pH
H1	4.03×10^{-5}	3.39
H2	8.18×10^{-5}	3.48
F	8.83×10^{-5}	3.54

Table S2. Winery data for total M3G concentration and pH for samples (S) of W_1 collected in the first half (H1), second half (H2), and final stage (F) of fermentation.

Modelled data describing pigmented anthocyanin species in W_1 throughout fermentation

S	Cond.	A^-	A	AH^+	$(A^-)_n$	$(A)_n$	$(AH^+)_n$	Pig. M3G
H1	a	3.01×10^{-12}	6.01×10^{-8}	1.90×10^{-6}	2.18×10^{-18}	1.11×10^{-8}	3.82×10^{-5}	4.02×10^{-5}
	b	1.58×10^{-6}	3.16×10^{-2}	1	1.15×10^{-12}	5.82×10^{-3}	20.08	
	c	5.60×10^{-10}	1.43×10^{-6}	5.84×10^{-6}	1.71×10^{-14}	3.08×10^{-6}	2.72×10^{-5}	3.76×10^{-5}
	d	9.59×10^{-5}	0.25	1	2.93×10^{-9}	0.53	4.66	
H2	a	3.16×10^{-12}	6.31×10^{-8}	1.99×10^{-6}	2.59×10^{-18}	1.34×10^{-8}	7.96×10^{-5}	8.17×10^{-5}
	b	1.58×10^{-6}	3.16×10^{-2}	1	1.30×10^{-12}	6.72×10^{-3}	39.88	
	c	6.28×10^{-10}	1.30×10^{-6}	4.30×10^{-6}	3.95×10^{-14}	1.32×10^{-5}	6.05×10^{-5}	7.93×10^{-5}
	d	1.46×10^{-4}	0.30	1	9.19×10^{-9}	3.07	14.07	
F	a	3.19×10^{-12}	6.36×10^{-8}	2.01×10^{-6}	2.65×10^{-18}	1.37×10^{-9}	8.61×10^{-5}	8.82×10^{-5}
	b	1.58×10^{-6}	3.16×10^{-2}	1	1.32×10^{-12}	6.81×10^{-3}	42.84	
	c	8.47×10^{-10}	1.52×10^{-6}	4.39×10^{-6}	6.86×10^{-14}	2.45×10^{-5}	5.50×10^{-5}	8.54×10^{-5}
	d	1.93×10^{-4}	0.35	1	1.56×10^{-8}	5.58	12.53	

Table S3. Concentrations and Gaussian parameters for samples (S) of W_1 collected in the first half (H1), second half (H2), and final stage (F) fermentation. For each pigmented anthocyanin species (A^-, A, AH^+, $(A^-)_n$, $(A)_n$, $(AH^+)_n$), data represent the (**condition a**) modelled concentrations (M) and the sum of these values (Pig. M3G) at the eluent pH, (**condition b**) Gaussian eluent concentration ratios (G_1) with respect to the monomeric flavylium cation AH^+, (**condition c**) modelled concentrations (M) and the sum of these values (Pig. M3G) at the wine sample pH, and (**condition d**) Gaussian wine sample concentration ratios (G_2) with respect to AH^+.

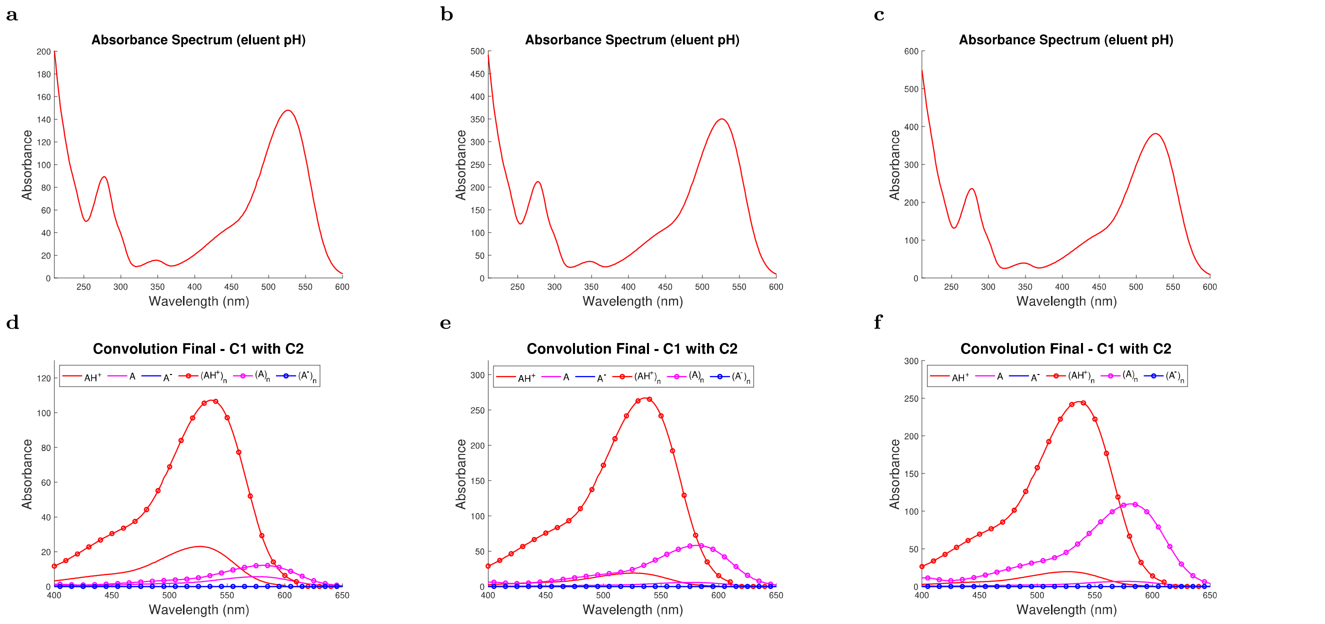

Figure S1. Top row, **a-c**, Experimental spectra in the form of the monomeric flavylium cation and (bottom row, **d**–**f**) corresponding systems of deconvoluted spectra for all pigmented anthocyanin species for samples of W_1 collected during fermentation in (**a**, **d**) first half (H1), (**b**, **e**) second half (H2), and (**c**, **f**) final stage (F).

Results showed that the systems of deconvoluted spectra (Figure S1d–f) provided much greater resolution than what was afforded by the flavylium-form experimental spectra on their own (Figure S1a–c), as they represented all pigmented anthocyanin species that exhibited varying colour hues, intensities, and levels of stability in solution[3,15,16] (eq. 3–9 of the main text and Supporting Information section 2). To characterise wine hues, experimental spectral data obtained using HPLC methods provide greater information (e.g., allowing for more accurate quantification of total anthocyanins in an automated fashion) compared to fundamental Vis spectral methods[17]. However, pH-controlled HPLC eluents still restrict accurate quantification of pH-dependent anthocyanin species (eq. 3–9 of the main text and Supporting Information section 2). This is represented for commercial fermentation samples shown in Figure 2 of the main text and Figure S1, as HPLC Vis spectra only represented M3G in the form of the red flavylium cation monomer (Figure S1a–c) due to acidic conditions that were required for analysis (Section 2.1 of the main text). In comparison, deconvoluted data revealed that flavylium cation monomers decreased in concentration between samples H1 (5.84×10^{-6} M) and H2 (4.30×10^{-6} M) (Table S3) as they were incorporated into self-associated complexes when total M3G concentration increased (i.e., 4.03×10^{-5} M for H1 to 8.18×10^{-5} M for H2, Table S2) and converted into quinonoidal base monomers that could self-associate when wine pH increased (i.e., 3.39 for H1 to 3.48 for H2, Table S2) (eq. 3–9 of the main text and Supporting Information section 2). The deconvoluted data also showed that the dark red self-associated flavylium cation ($(AH^+)_n$) was the most abundant species in all samples (Figure S1d–f)[3], and that amounts of the purple monomeric quinonoidal base and dark purple self-associated quinonoidal base species increased between samples H1 to F (Table S3 and Figure S1d–f) as wine pH increased (i.e., 3.39 for H1 to 3.54 for F, Table S2).

Regarding colour intensity, experimental measurements between samples H1 to F demonstrated increases in total M3G concentration (i.e., 4.03×10^{-5} M for H1 to 8.83×10^{-5} M for F, Table S2) and

spectral absorbances (Figure S1a–c) as phenolics were extracted from grape into wine throughout macerative fermentation, causing the wines to become darker in colour[18,9]. The modelled deconvolution system (Figure S1d–f) provided increased information compared to experimental data (Figure S1a–c) about the colour intensity of samples by representing behaviours of self-associated pigmented anthocyanin species (i.e., $(A^{-})_n$, $(A)_n$, $(AH^{+})_n$) whose formations were positively influenced by increases of total concentration (Table S2, Table S3, and Figure S1d–f). Summed concentrations of pigmented species in the samples (i.e., 3.76×10^{-5} M for H1, 7.93×10^{-5} M for H2, 8.54×10^{-5} M for F, Table S3) were shown to be slightly smaller (by an average of 2.70×10^{-6} M) than the experimentally measured values for total M3G concentration (i.e., 4.03×10^{-5} M for H1, 8.18×10^{-5} M for H2, 8.83×10^{-5} M for F, Table S2) and may be more accurate indicators for predicting colour and stability characteristics of samples, as they accounted solely for pigmented anthocyanin species. Knowledge of spectral features provided by the model (Section 2.2 of the main text and Supporting Information sections 1–3) could be useful for controlling and stabilising anthocyanins in natural dyes, pH-indicators, and other anthocyanin-containing products (e.g., food, beverages) that possess acidic conditions[17,19,20,21,22]. Overall, results indicated that traditional HPLC-DAD Vis spectra for anthocyanins (Figure S1a–c) may provide important "hidden" information about the anthocyanin multistate system that deconvolution can reveal (Figure S1d–f). Comparisons of colourimetric data between experimental and deconvoluted systems is provided in Supporting Information section 5.

4.2. Commercial Fermentations W_2 and W_3

Further evidence of applications of the developed deconvolution model (Section 2.2 of the main text and Supporting Information sections 1–3) for red wine analysis (Section 3.1 of the main text and Supporting Information section 4.1) are demonstrated with additional commercial Shiraz fermentations, W_2 and W_3. Details for sample collection and analysis are described in Section 2.1 of the main text, and samples collected at fermentation stages SI, H1, H2, and F are considered here (i.e., Table S4, Table S5, and Figure S2 for W_2, Table S6, Table S7, and Figure S3 for W_3).

Winery data for W_2 throughout fermentation

S	Total M3G Concentration (M)	pH
SI	9.02×10^{-5}	3.44
H1	1.41×10^{-4}	3.51
H2	1.34×10^{-4}	3.61
F	1.42×10^{-4}	3.66

Table S4. HPLC data for total M3G concentration (M) and empirical data for pH of samples (S) SI, H1, H2, and F of W_2.

Modelled data describing pigmented anthocyanin species in W_2 throughout fermentation

S	Cond.	A^{-}	A	AH^{+}	$(A^{-})_n$	$(A)_n$	$(AH^{+})_n$	Pig. M3G
SI	a	3.19×10^{-12}	6.37×10^{-8}	2.01×10^{-6}	2.66×10^{-18}	1.38×10^{-8}	8.79×10^{-5}	9.00×10^{-5}
	b	1.58×10^{-6}	3.16×10^{-2}	1	1.32×10^{-12}	6.84×10^{-3}	43.73	
	c	4.65×10^{-10}	1.05×10^{-6}	3.80×10^{-6}	2.66×10^{-14}	1.00×10^{-5}	7.33×10^{-5}	8.82×10^{-5}
	d	1.22×10^{-4}	0.28	1	7.00×10^{-9}	2.63	19.29	
H1	a	3.33×10^{-12}	6.65×10^{-8}	2.10×10^{-6}	2.97×10^{-18}	1.56×10^{-8}	1.39×10^{-4}	1.41×10^{-4}
	b	1.58×10^{-6}	3.16×10^{-2}	1	1.41×10^{-12}	7.40×10^{-3}	66.19	

	c	5.55×10^{-10}	1.08×10^{-6}	3.33×10^{-6}	4.64×10^{-14}	3.19×10^{-5}	1.03×10^{-4}	1.39×10^{-4}
	d	1.67×10^{-4}	0.32	1	1.39×10^{-8}	9.58	30.93	
H2	a	3.31×10^{-12}	6.61×10^{-8}	2.09×10^{-6}	2.93×10^{-18}	1.53×10^{-8}	1.32×10^{-4}	1.34×10^{-4}
	b	1.58×10^{-6}	3.16×10^{-2}	1	1.40×10^{-12}	7.33×10^{-3}	63.16	
	c	9.03×10^{-10}	1.39×10^{-6}	3.43×10^{-6}	1.04×10^{-13}	7.11×10^{-5}	5.53×10^{-5}	1.31×10^{-4}
	d	2.63×10^{-4}	0.41	1	3.03×10^{-8}	20.73	16.12	
F	a	3.33×10^{-12}	6.64×10^{-8}	2.10×10^{-6}	2.97×10^{-18}	1.56×10^{-8}	1.40×10^{-4}	1.42×10^{-4}
	b	1.58×10^{-6}	3.16×10^{-2}	1	1.42×10^{-12}	7.41×10^{-3}	66.67	
	c	1.04×10^{-9}	1.42×10^{-6}	3.11×10^{-6}	1.42×10^{-13}	9.52×10^{-5}	4.00×10^{-5}	1.40×10^{-4}
	d	3.34×10^{-4}	0.46	1	4.57×10^{-8}	30.61	12.86	

Table S5. Modelled concentrations of pigmented anthocyanin species and Gaussian parameters for samples SI, H1, H2, and F of W_2, taking the total M3G concentration (M) and pH values displayed in Table 4. Conditions **a**–**d** are the same as those presented in Table S3.

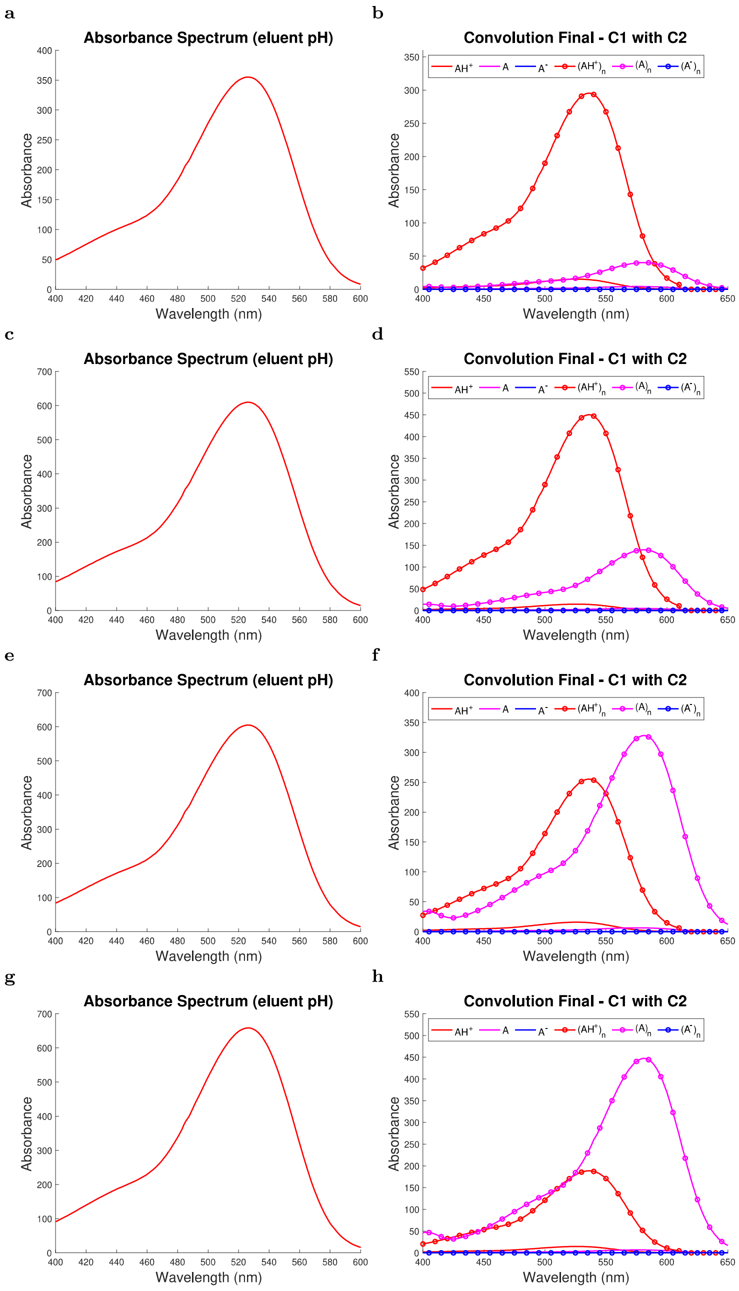

Figure S2. Left column, **a**,**c**,**e**,**g**, Experimental spectra in the form of the monomeric flavylium cation and (right column, **b**,**d**,**f**,**h**) corresponding systems of deconvoluted spectra for all pigmented anthocyanin species for samples of W_2 collected during (**a**,**b**) initial (SI), (**c**,**d**) first half (H1), (**e**,**f**) second half (H2), and (**g**,**h**) final (F) stages of fermentation.

Winery data for W_3 throughout fermentation

S	Total M3G Concentration (M)	pH
SI	4.11×10^{-5}	3.34
H1	9.22×10^{-5}	3.57
H2	9.48×10^{-5}	3.62
F	1.08×10^{-4}	3.65

Table S6. HPLC data for total M3G concentration (M) and empirical data for pH of samples (S) SI, H1, H2, and F of W_3.

Modelled data describing pigmented anthocyanin species in W_3 throughout fermentation

S	Cond.	A^-	A	AH^+	$(A^-)_n$	$(A)_n$	$(AH^+)_n$	Pig. M3G
SI	a	3.02×10^{-12}	6.02×10^{-8}	1.90×10^{-6}	2.19×10^{-18}	1.11×10^{-8}	3.90×10^{-5}	4.10×10^{-5}
	b	1.58×10^{-6}	3.16×10^{-2}	1	1.15×10^{-12}	5.84×10^{-3}	20.53	
	c	3.88×10^{-10}	1.10×10^{-6}	5.06×10^{-6}	1.05×10^{-14}	2.19×10^{-6}	3.06×10^{-5}	3.90×10^{-5}
	d	7.67×10^{-5}	0.22	1	2.08×10^{-9}	0.43	6.05	
H1	a	3.19×10^{-12}	6.36×10^{-8}	2.01×10^{-6}	2.67×10^{-18}	1.38×10^{-8}	9.00×10^{-5}	9.21×10^{-5}
	b	1.58×10^{-6}	3.16×10^{-2}	1	1.33×10^{-12}	6.87×10^{-3}	44.78	
	c	9.61×10^{-10}	1.61×10^{-6}	4.34×10^{-6}	8.69×10^{-14}	3.30×10^{-5}	5.02×10^{-5}	8.92×10^{-5}
	d	2.21×10^{-4}	0.37	1	2.00×10^{-8}	7.60	11.57	
H2	a	3.22×10^{-12}	6.43×10^{-8}	2.03×10^{-6}	2.70×10^{-18}	1.40×10^{-8}	9.26×10^{-5}	9.47×10^{-5}
	b	1.58×10^{-6}	3.16×10^{-2}	1	1.33×10^{-12}	6.90×10^{-3}	45.62	
	c	1.20×10^{-10}	1.81×10^{-6}	4.34×10^{-6}	1.27×10^{-13}	4.67×10^{-5}	3.85×10^{-5}	9.14×10^{-5}
	d	2.76×10^{-5}	0.42	1	2.93×10^{-8}	10.76	8.87	
F	a	3.24×10^{-12}	6.47×10^{-8}	2.05×10^{-6}	2.78×10^{-18}	1.45×10^{-8}	1.06×10^{-4}	1.08×10^{-4}
	b	1.58×10^{-6}	3.16×10^{-2}	1	1.36×10^{-12}	7.07×10^{-3}	51.71	
	c	1.20×10^{-10}	1.69×10^{-6}	3.77×10^{-6}	1.47×10^{-13}	6.41×10^{-5}	3.55×10^{-5}	1.05×10^{-4}
	d	3.18×10^{-5}	0.45	1	3.90×10^{-8}	17.00	9.42	

Table S7. Modelled concentrations of pigmented anthocyanin species and Gaussian parameters for samples SI, H1, H2, and F of W_3, taking the total M3G concentration (M) and pH values displayed in Table 4. Conditions **a**–**d** are the same as those presented in Table S3.

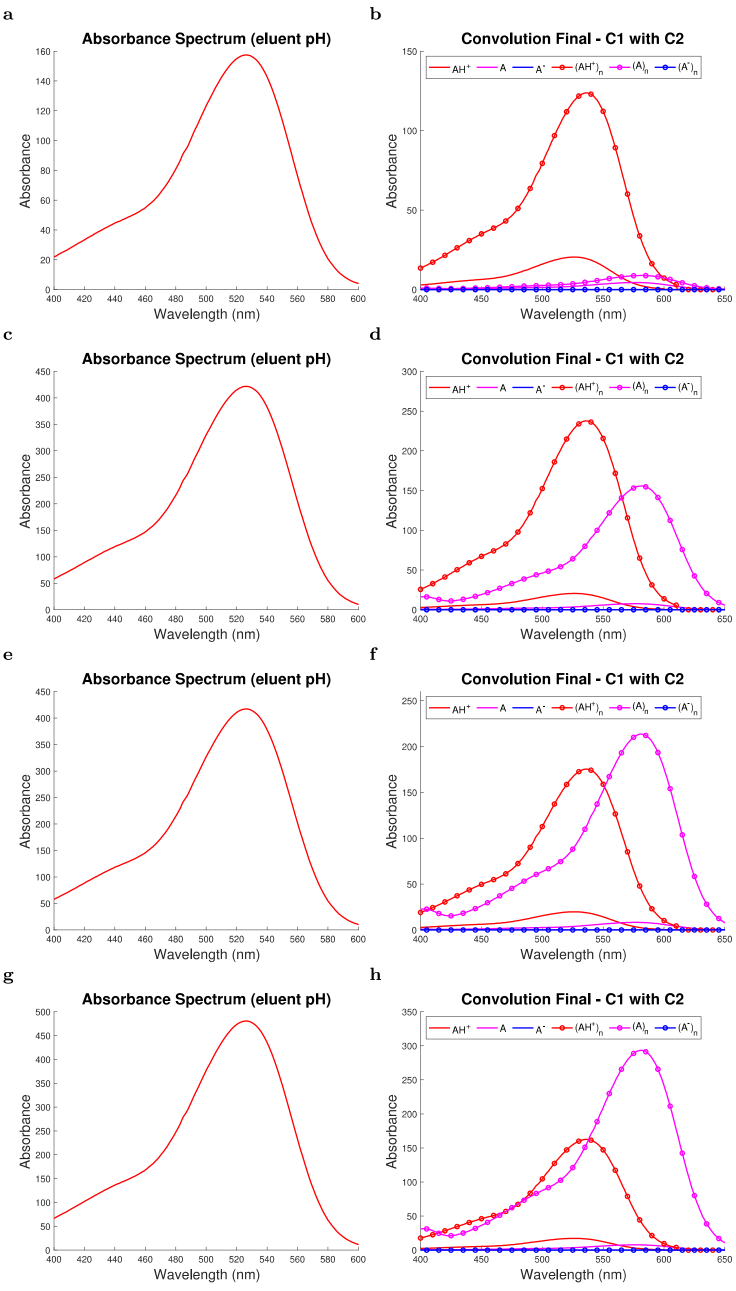

Figure S3. Left column, **a**,**c**,**e**,**g**, Experimental spectra in the form of the monomeric flavylium cation and (right column, **b**,**d**,**f**,**h**) corresponding systems of deconvoluted spectra for all pigmented anthocyanin species for samples of W_3 collected during (**a**,**b**) initial (SI), (**c**,**d**) first half (H1), (**e**,**f**) second half (H2), and (**g**,**h**) final (F) stages of fermentation.

Simulated results indicated that the deconvoluted spectra for W_2 (Figure S2b,d,f,h) and W_3 (Figure S3b,d,f,h) reveal greater information about the anthocyanin multistate system (eq. 3–9 of the main text and Supporting Information section 2) than is afforded by HPLC data for W_2 (Figure S2a,c,e,g) and W_3 (Figure S3a,c,e,g) on their own. Both systems display pH-dependent behaviours (i.e., amounts of A, AH^+) and self-association that is dependent on M3G concentration (i.e., amounts of $(A)_n$, $(AH^+)_n$) that provide essential pigments and colour stability attributes to the commercial fermentations (i.e., W_2, W_3).

Key insights from the deconvoluted data for both of these (i.e., Figure S2b,d,f,h for W_2 and Figure S3b,d,f,h for W_3) showed that amounts of purple quinonoidal base species (i.e., A, $(A)_n$) increased between samples SI–F as pH increased (i.e., Table S4 for W_2 and Table S6 for W_3). Concentrations of pigmented species (i.e., Table S5 for W_2 and Table S7 for W_3) also increased as total M3G concentration increased (i.e., between SI to H1 and H2 to F for W_2, Table S4 and between SI to F for W_3, Table S6).

In particular, the deconvoluted system of W_3 (Figure S3b,d,f,h) exhibited increases in concentration of the self-associated quinonoidal base $(A)_n$ between wine samples SI and F (i.e., from 2.19×10^{-6} M to 6.41×10^{-6} M, Table S7, condition c) as wine pH and total M3G concentration increased (Table S6). As a result, the multistate system's equilibrium shifted away from formation of the monomeric flavylium species AH^+ (i.e., with the concentration of AH^+ decreasing from 5.06×10^{-6} M in SI to 3.77×10^{-6} M in F, Table S7, condition c). Notably, this is in contrast to information provided by the flavylium-form HPLC data (Figure S3a,c,e,g), which shows an increase in M3G absorbance that is reflective of increases in total M3G concentration that occur throughout fermentation (i.e., from 4.11×10^{-5} M in SI to 1.08×10^{-4} M in F, Table S6). These results indicate that winery data may occasionally indicate that a sample is increasing in red hues (afforded by the monomeric flavylium cation under acidic eluent conditions used in laboratory analysis), when in actuality, the sample colour is being modified and even lessening in red hues as the flavylium cation undergoes reactions with other anthocyanin species (influenced by parameters including pH, total anthocyanin concentration, and temperature)[9,3] in the sample. These findings indicate that there is a need for computational methods to accurately represent behaviours of reactive anthocyanins within analysed samples, as is presented in the current work (Section 2.2 of the main text and Supporting Information sections 1–3).

5. Colourimetry

5.1. Colourimetry Method

For the modelled output, RGB colour values were chosen to represent each of the monomeric and self-associated anthocyanin species, with choices informed by experimental wine sample data[3]. The RGB coordinates for each wine sample were determined using the expression:

$$C_{R,G,B} = \sum_{i=1}^{6} S_{\%} S_{R,G,B} \quad (S32)$$

where $C_{R,G,B}$ is one of the determined red, green, or blue colour values, and i enumerates the six considered pigmented anthocyanin species (i.e., A^-, A, AH^+, $(A^-)_n$, $(A)_n$ and $(AH^+)_n$). Then $S_{\%}$ is the modelled percent ratio for a given anthocyanin species with respect to the summed M3G concentration of all pigmented species. Further, $S_{R,G,B}$ is the corresponding assigned red, green, or blue colour value for a given anthocyanin species, with values modified slightly from those reported previously[3] to account for the absence of the colourless hemiketal within the present deconvolution system (e.g., where data are transformed for pigmented species in the visible region of the absorbance spectrum). As such, RGB values are assigned as (151, 195, 194) for the blue quinonoidal anion A^-, (176, 164, 185) for the purple quinonoidal base A, (199, 75, 103) for the red flavylium cation AH^+, (15, 35, 170) for the dark blue self-associated quinonoidal anion $(A^-)_n$, (92, 35, 108) for the dark purple self-associated quinonoidal base $(A)_n$ and (154, 35, 31) for the dark red self-associated flavylium cation $(AH^+)_n$.

5.2. Colourimetric Profiles for W_1

To provide visual representations of the presented spectral data of W_1 (Figure 2 of the main text and Figure S1), concentrations of all pigmented anthocyanin species were assigned colour coordinates using the method presented in Supporting Information section 5.1. Colour values were then calculated for the displayed experimental and deconvoluted data for acidic (e.g., red wine) samples (Figure 2 of the main text and Figure S1) based on the anthocyanin species profiles for each system (i.e., Table 1 of the main text and Table S3, respectively). Resultant colour profiles are visualised in Figure S4 with values provided in Table S8.

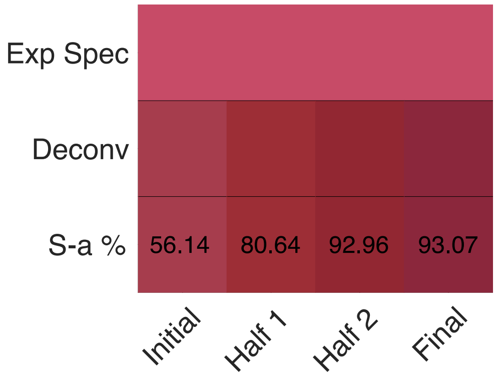

Figure S4. Colourimetric profiles for the spectral data presented for the samples collected in initial (SI) (Figure 2 of the main text), first half (H1), second half (H2), and final (F) (Figure S1) stages of fermentation for W_1. Profiles represent the experimentally collected (HPLC-DAD) Vis spectra for M3G (Exp Spec), systems of deconvoluted spectra for all pigmented anthocyanin species (Deconv), and percentages of self-associated species (i.e., $(A^-)_n + (A)_n + (AH^+)_n$) out of all pigmented species (i.e., $A^- + A + AH^+ + (A^-)_n + (A)_n + (AH^+)_n$) (S-a%).

Simulated RGB coordinates for samples of W_1 throughout fermentation

	Exp			Md		
S	C_R	C_G	C_B	C_R	C_G	C_B
SI	199	75	103	166	61	77
H1	199	75	103	157	46	54
H2	199	75	103	146	39	50
F	199	75	103	139	39	60

Table S8. Red (C_R), green (C_G), and blue (C_B) colourimetric coordinates for the HPLC (Exp) and deconvoluted (Md) spectral colour profiles shown in Figure S4.

Colour profiles for samples of commercial red wine ferment W_1 (Figure S4) showed that the experimentally generated spectra (Figure 2a of the main text and Figure S1a–c), which were in the monomeric flavylium cation form due to eluent pH (Section 2.1), corresponded with red hues in all samples. The corresponding deconvoluted spectra (Figure 2e of the main text and Figure S1d–f) corresponded with darker hues (Figure S4) which appeared pink in sample SI when the summed pigmented species concentration was at its lowest (i.e., 1.98×10^{-5} M, Table 1 of the main text), dark red in samples H1 and H2 as the concentration increased (i.e., to 3.76×10^{-5} M for H1 and 7.93×10^{-5} M for H2, Table S3), and dark purple in sample F as the concentration increased again slightly (i.e., to

8.54 × 10^{-5} M, Table S3) and solution pH rose (attaining a value of 3.54 for F, Table S2), generating formation of the quinonoidal base species (eq. 3–9 of the main text)[23,24]. This visualisation (Figure S4) reflects claims presented earlier (Section 3.1 of the main text and Supporting Information section 4) that the developed deconvolution model (Section 2.2 of the main text and Supporting Information sections 1–3) provides greater information about colour hue and intensity characteristics for acidic anthocyanin-containing solutions than is available from experimental HPLC data alone (Figure 2 of the main text and Figure S1).

Percentages of self-associated species in solution can also be generated by the model (Figure S4), where percentages increased largely between samples SI and H2 (i.e., from 56.14% to 92.96%) and then minorly between samples H2 and F (i.e., from 92.96% to 93.07%) of W_1, as phenolic extraction caused total M3G concentration to rise and eventually plateau throughout fermentation (Table 1 of the main text and Table S2)[18]. Such results could provide meaningful insights about the stability of anthocyanins within solution (Figure S4)[2,11,16] that are not attainable from traditional experimental methods alone (Figure 2 of the main text and Figure S1), as shown previously for results under acidic conditions (Section 3.1 of the main text and Supporting Information section 4).

5.3. Colourimetry with Concentration-Dependent Coordinates

The red wine colour profiles for W_1 (Supporting Information section 5.2) were then visualised using colour coordinates that vary with respect to the concentration of each pigmented anthocyanin species, which represented how sample colours would darken between SI and F as concentrations of pigmented species (Table 1 of the main text and Table S3) and corresponding Vis spectral absorbances of M3G (Figure 2a of the main text and Figure S1a–c) increased. Values are linearly dependent on the summed M3G concentrations (M) of all pigmented species for samples SI (Figure 1 of the main text), H1, H2, and F (Table S3) of W_1, taking the largest of these (8.54 × 10^{-5} M) to have the colour coordinates assigned in Supporting Information section 5.1.

Concentration-dependent RGB coordinates for pigmented anthocyanin species

Pig. M3G	RGB	A^-	A	AH^+	$(A^-)_n$	$(A)_n$	$(AH^+)_n$
1.98 × 10^{-5}	C_R	230	236	242	199	217	231
	C_G	241	233	213	203	203	203
	C_B	240	238	219	235	220	203
3.76 × 10^{-5}	C_R	209	220	230	149	183	210
	C_G	228	214	175	158	158	158
	C_B	228	224	188	217	190	156
7.93 × 10^{-5}	C_R	158	181	203	32	103	161
	C_G	199	170	87	50	50	50
	C_B	198	190	113	176	118	47
8.54 × 10^{-5}	C_R	151	176	199	15	92	154
	C_G	195	164	75	35	35	35
	C_B	194	185	103	170	108	31

Table S9. Red (C_R), green (C_G), and blue (C_B) colourimetric coordinates for pigmented anthocyanin species (i.e., A^-, A, AH^+, $(A^-)_n$, $(A)_n$ and $(AH^+)_n$) that are dependent on M3G concentration (M) of each species.

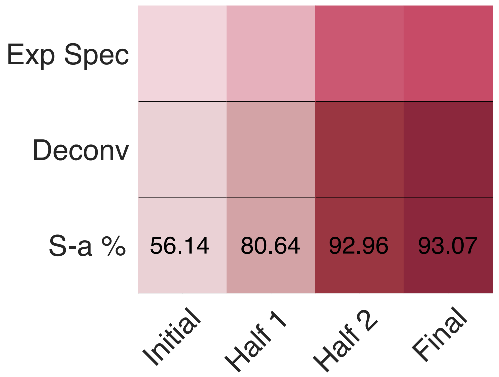

Figure S5. The red wine profiles presented in Figure S4 are visualised here using the concentration-dependent colour coordinates provided in Table S9.

Simulated concentration-dependent RGB coordinates for samples of W_1 throughout fermentation

	Exp			Md		
S	C_R	C_G	C_B	C_R	C_G	C_B
SI	242	213	220	234	209	213
H1	230	176	188	211	163	166
H2	203	88	114	154	54	65
F	199	75	103	139	39	60

Table S10. Red (C_R), green (C_G), and blue (C_B) colourimetric coordinates for the HPLC (Exp) and deconvoluted (Md) spectral colour profiles shown in Figure S5, which are derived using concentration-dependent colour values for each pigmented anthocyanin species.

Colour profiles for experimental spectra (Figure S5, condition Exp Spec) were then able to show that while these systems contained only AH^+ and not self-associated species (Figure 2a of the main text and Figure S1a–c), they increased in darkness between samples SI and F (Figure S5) as total M3G concentration, determined by HPLC, increased (Table 1 of the main text and Table S2). Results showed that colour profiles predicted by the deconvolution model (Figure S5, condition Deconv) would not vary significantly from those for experimental spectra in samples SI and H1 (Figure S5, condition Exp Spec) when using the considered colourimetric coordinates (Table S10); however, the deconvoluted system still provided information about self-associated species percentages (Figure S5, condition S-a %) that were not available from HPLC-DAD spectral data alone (Figure S5, condition Exp Spec).

Colour profiles for the deconvolution model then exhibited greater variations than experimental spectra in samples H2 and F (Figure S5, condition Deconv), as pigmented species concentrations increased (Table 1 of the main text and Table S3) and the dark red and dark purples hues afforded by the self-associated species in the deconvoluted system (i.e., $(AH^+)_n$ and $(A)_n$, respectively) (Figure S1e,f) were revealed. Regardless of the colourimetric coordinates used (as provided in Supporting Information section 5.1 for Figure S4 and Supporting Information section 5.3 for Figure S5), the abilities demonstrated by the developed model to describe colour and stability characteristics of acidic

anthocyanin-containing solutions (Figures S4 and S5) is a meaningful outcome that could have benefits for the analysis of extracts, dyes, pH-indicators, and red wines[20,19,17,25].

6. Spectral Deconvolution in Neutral and Basic Simulations

6.1. Modelled Gaussian Parameters for all Pigmented Species

Modelled parameters (Supporting Information section 2) corresponding with the deconvoluted simulations shown in Figure 3 of the main text are provided in Table S11. Simulated samples possessed a total M3G concentration of 2.34×10^{-5} M, with samples and eluents assigned to have equal pH values under neutral (5.0, 6.5) and basic (8.0, 9.0) conditions. Values for equilibrium (J) and kinetic rate ($j_{\pm}$) constants were chosen to reflect anthocyanin self-association behaviours in pure solutions (Supporting Information section 2)[8,2] where interactions are not inhibited by heat and ethanol[4].

Modelled concentrations of pigmented anthocyanin species in neutral and basic media

pH	Cond.	A^-	A	AH^+	$(A^-)_n$	$(A)_n$	$(AH^+)_n$	Pig. M3G
5.0	Pa	6.57×10^{-8}	4.14×10^{-6}	4.14×10^{-7}	6.99×10^{-11}	1.08×10^{-5}	1.81×10^{-8}	1.54×10^{-5}
	Pb	1.58×10^{-2}	1	0.10	1.69×10^{-5}	2.60	4.37×10^{-3}	
6.5	Pa	1.94×10^{-6}	3.88×10^{-6}	1.23×10^{-8}	6.69×10^{-8}	1.00×10^{-5}	1.64×10^{-11}	1.59×10^{-5}
	Pb	0.50	1	3.16×10^{-3}	1.72×10^{-2}	2.58	4.23×10^{-6}	
8.0	Pa	7.75×10^{-6}	4.89×10^{-7}	4.89×10^{-11}	1.35×10^{-5}	6.81×10^{-7}	1.55×10^{-15}	2.25×10^{-5}
	Pb	1	6.31×10^{-2}	6.31×10^{-6}	1.75	8.79×10^{-2}	1.99×10^{-10}	
9.0	Pa	3.80×10^{-6}	2.40×10^{-8}	2.40×10^{-13}	1.95×10^{-5}	2.80×10^{-9}	1.15×10^{-19}	2.34×10^{-5}
	Pb	1	6.31×10^{-3}	6.31×10^{-8}	5.14	7.37×10^{-4}	3.03×10^{-14}	

Table S11. Modelled values represent the (**condition Pa**) concentrations (M) of all pigmented monomeric (A^-, A, AH^+) and self-associated ($(A^-)_n$, $(A)_n$, $(AH^+)_n$) anthocyanin species at the pH of the eluent and sample, with total M3G concentration being 2.34×10^{-5} M, and (**condition Pb**) Gaussian concentration ratios for the eluent and sample ($G_1 = G_2$) of all species with respect to the monomeric quinonoidal base A at neutral pH values (pH = 5.0 and 6.5) and monomeric quinonoidal anion A^- in alkaline media (pH = 8.0 and 9.0).

6.2. Colourimetry for Neutral and Basic Spectra

Colour profiles were generated for the presented hypothetical HPLC-DAD Vis spectra and corresponding deconvoluted systems for solutions possessing neutral (Figure 3a–d of the main text and Table S11) and basic (Figure 3e–h of the main text and Table S11) pH conditions. Using the colourimetric coordinates presented in Supporting Information section 5.1, colour profiles for each solution are visualised in Figure S6 with values provided in Table S12.

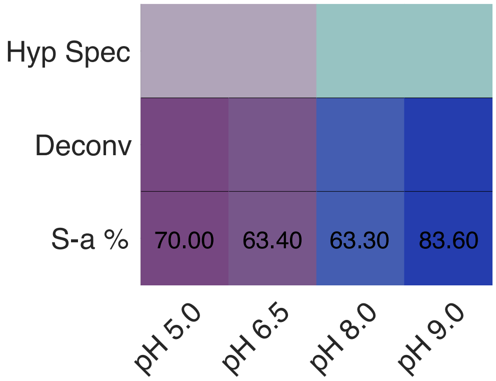

Figure S6. Colourimetric profiles for the spectral data presented in Figure 3 of the main text for neutral and basic pH samples. Profiles represent hypothetical HPLC-DAD Vis spectra for M3G (Hyp Spec), systems of deconvoluted spectra for all pigmented anthocyanin species (Deconv), and percentages of self-associated species (i.e., $(A^{-})_n + (A)_n + (AH^{+})_n$) out of all pigmented species (i.e., $A^{-} + A + AH^{+} + (A^{-})_n + (A)_n + (AH^{+})_n$) (S-a%) for samples and corresponding eluents with pH values of 5.0, 6.5, 8.0, and 9.0.

Simulated RGB coordinates for hypothetical solutions possessing neutral and basic pH values

	Exp			Md		
pH	C_R	C_G	C_B	C_R	C_G	C_B
5.0	176	164	185	118	71	129
6.5				119	86	138
8.0	151	195	194	68	93	177
9.0				37	61	174

Table S12. Red (C_R), green (C_G), and blue (C_B) colourimetric coordinates for the HPLC (Exp) and deconvoluted (Md) spectral colour profiles shown in Figure S6.

Simulations for hypothetical spectra showed that solutions with neutral pH values of 5.0 (Figure 3a of the main text) and 6.5 (Figure 3c of the main text) that are comprised of the quinonoidal base would be indicated by HPLC Vis data to display light purple hues (Figure S6), while HPLC Vis data would indicate that alkaline solutions with pH values of 8.0 (Figure 3e of the main text) and 9.0 (Figure 3g of the main text) that are comprised of the quinonoidal anion would display light blue hues (Figure S6). This is owed to the fact that all pigmented species of M3G would get rapidly converted into the respective species for each eluent pH condition (i.e., A with pH conditions of 5.0 and 6.5, A^{-} when pH values are 8.0 and 9.0) during HPLC analysis (eq. 3–9 and Supporting Information section 2)[23,24].

In comparison, data generated by the developed deconvolution model would provide greater resolution for displaying key colour characteristics afforded by anthocyanins. Dark purple and dark blue hues were observable in colour profiles for the deconvoluted spectra (Figure S6), with colour shades varying with respect to each system's concentrations of self-associated species (i.e., $(A)_n$ at pH 5.0 (Figure 3b of the main text) and 6.5 (Figure 3d of the main text), $(A)_n$ and $(A^{-})_n$ at pH 8.0 (Figure 3f of the main text), and $(A^{-})_n$ at pH 9.0 (Figure 3h of the main text)). The model also revealed that self-

associated species would have a greater presence in solutions with pH values of 5.0 (70.00%) and 9.0 (83.60%), compared to with pH values of 5.0 (63.40%) and 9.0 (63.30%) (Figure S6), as the dominating monomers at pH 5.0 (A) and 9.0 (A^{-}) would not be in major competition with other pigmented species at these pH values (Supporting Information section 2) and would therefore highly available for self-association (Figure 3b of the main text for pH 5.0 and Figure 3h of the main text for pH 9.0).

Capabilities of the developed deconvolution model (Section 2.2 of the main text and Supporting Information sections 1–3) to generate spectra and colourimetric data for neutral and basic pH solutions (Section 3.2 of the main text and Supporting Information section 6) may have applications for increasing identification and stabilisation of purple (A, $(A)_n$) and blue (A^{-}, $(A^{-})_n$) anthocyanin species in products such as dyes, food, and pH-sensors[26,27,28,20,19]. Such results could be especially relevant in research and industry settings to increase application of blue-purple anthocyanins (e.g., from elderberries, purple sweet potatoes, butterfly pea flowers) that contain high amounts of these species[28,21,29].

References

1. Muhlack, R. MATLAB code to model anthocyanin proton transfer, hydration and self-association. figshare https://doi.org/10.25909/25001540.v2 (2024).
2. Tindal, R. A., Jeffery, D. W., & Muhlack, R. A. (2024a). Nonlinearity and anthocyanin colour expression: A mathematical analysis of anthocyanin association kinetics and equilibria. *Food Research International, 183*, 114195.
3. Tindal, R. A., Jeffery, D. W., & Muhlack, R. A. (2024b). Mathematical modelling and chemical analysis to characterise anthocyanin self-association interactions influencing colour expression and stability in young red wines. *Food and Bioprocess Technology (In press).*
4. Tseng, K.-C., Chang, H.-M., & Wu, J. S.-B. (2006). Degradation kinetics of anthocyanin in ethanolic solutions. *Journal of Food Processing and Preservation, 30*(5), 503–514.
5. Sadlowski, E. (1985). *pH-Dependent anthocyanin reactions in micellar and copigmented solutions* [Doctoral thesis, Colorado State University].
6. Moreira, P. F., Giestas, L., Yihwa, C., Vautier-Giongo, C., Quina, F. H., Maçanita, A. L., & Lima, J. C. (2003). Ground- and excited-state proton transfer in anthocyanins: From weak acids to superphotoacids. *Journal of Physical Chemistry A, 107*(21), 4203–4210.
7. Brouillard, R., & Lang, J. (1990). The hemiacetal-*cis*-chalcone equilibrium of malvin, a natural anthocyanin. *Canadian Journal of Chemistry, 68*(5), 755–761.
8. Hoshino, T. (1992). Self-association of flavylium cations of anthocyanidin 3,5-diglucosides studied by circular dichroism and ^{1}H NMR. *Phytochemistry, 31*(2), 647–653.
9. He, F., Liang, N.-N., Mu, L., Pan, Q.-H., Wang, J., Reeves, M. J., & Duan, C.-Q. (2012). Anthocyanins and their variation in red wines I. Monomeric anthocyanins and their color expression. *Molecules, 17*(2), 1571–1601.
10. Han, F. L., & Xu, Y. (2015). Effect of the structure of seven anthocyanins on self-association and colour in an aqueous alcohol solution. *South African Journal of Enology and Viticulture, 36*(1), 105–116.
11. Escribano-Bailón, M. T., Rivas-Gonzalo, J. C., & García-Estévez, I. (2019). Wine color evolution and stability. In A. Morata (Ed.), *Red Wine Technology*, (pp. 195–205). Cambridge, MA, USA: Academic Press.
12. Lisandru, T.-T., Bunea, A., Füstös, A., Dumitraş, A., Bunea, C.-I., Dan, V. S., Gál, E., & Viorel, M. (2018). Chromatographic analysis of carotenoids and anthocyanins in sweet cherry autumn leaves used in ornamental landscapes. *Studia Universitatis Babes-Bolyai Chemia, 63*(2), 143–155.
13. Alejo-Armijo, A., Parola, A. J., & Pina, F. (2019). pH-dependent multistate system generated by a synthetic furanoflavylium compound: An ancestor of the anthocyanin multistate of chemical species. *ACS Omega, 4*(2), 4091–4100.
14. Alejo-Armijo, A., Mendoza, J., Parola, A. J., & Pina, F. (2020). Chemical evolution of the colour systems generated by riccionidin A, 3–deoxyanthocyanidins and anthocyanins. *Phytochemistry, 174*, 112339.
15. Petrov, V., & Pina, F. (2010). Analytical resolution of the reaction rates of flavylium network by Laplace transform. *Journal of Mathematical Chemistry, 47*, 1005–1026.

16. Gonzàlez-Manzano, S., Santos-Buelga, C., Dueñas, M., Rivas-Gonzalo, J. C., & Escribano-Bailón, T. (2008). Colour implications of self-association processes of wine anthocyanins. *European Food Research and Technology, 226*, 483–490.

17. Welch, C. R., Wu, Q., & Simon, J. E. (2008). Recent advances in anthocyanins analysis and characterization. *Current Analytical Chemistry, 4*(2), 75–101.

18. Setford, P. C., Jeffery, D. W., Grbin, P. R., & Muhlack, R. A. (2017). Factors affecting extraction and evolution of phenolic compounds during red wine maceration and the role of process modelling. *Trends in Food Science and Technology, 69*(Part A), 106-117.

19. Mulyaningsih, R. D., Pratiwi, R., & Hasanah, A. N. (2023). An update on the use of natural pigments and pigment nanoparticle adducts for metal detection based on colour response. *Biosensors, 13*(5), 554.

20. Tang, B., He, Y., Liu, J., Zhang, J., Li, J., Zhou, J., Ye, Y., Wang, J., & Wang, X. (2019). Kinetic investigation into pH-dependent color of anthocyanin and its sensing performance. *Dyes and Pigments, 170*, 107643.

21. Rodríguez-Mena, A., Ochoa-Martínez, L. A., González-Herrera, S. M., Rutiaga-Quiñones, O. M., González-Laredo, R. F., Olmedilla-Alonso, B., & Vega-Maturino, S. (2023). Coloring potential of anthocyanins from purple sweet potato paste: Ultrasound-assisted extraction, enzymatic activity, color and its application in ice pops. *Food Chemistry Advances, 3*, 100358.

22. Câmara, J. S., Locatelli, M., Pereira, J. A. M., Oliveira, H., Arlorio, M., Fernandes, I., Perestrelo, R., Freitas, V., & Bordiga, M. (2022). Behind the scenes of anthocyanins – from the health benefits to potential applications in food, pharmaceutical and cosmetic fields. *Nutrients, 14*(23), 5133.

23. Dangles, O., & Fenger, J. A. (2018). The chemical reactivity of anthocyanins and its consequences in food science and nutrition. *Molecules, 23*(8), 1970.

24. Basílio, N., & Pina, F. (2016). Chemistry and photochemistry of anthocyanins and related compounds: A thermodynamic and kinetic approach. *Molecules, 21*(11), 1502.

25. de Villiers, A., Vanhoenacker, G., Majek, P., & Sandra, P. (2004). Determination of anthocyanins in wine by direct injection liquid chromatography-diode array detection-mass spectrometry and classification of wines using discriminant analysis. *Journal of Chromatography A, 1054*(1–2), 195–204.

26. Rose, P. M., Cantrill, V., Benohoud, M., Tidder, A., Rayner, C. M., & Blackburn, R. S. (2018). Application of anthocyanins from blackcurrant (*Ribes nigrum* L.) fruit waste as renewable hair dyes. *Journal of Agricultural and Food Chemistry, 66*(26), 6790–6798.

27. Dangles, O. (2024). Anthocyanins as natural food colorings: The chemistry behind and challenges still ahead. *Journal of Agricultural and Food Chemistry, 72*(22), 12356–12372.

28. da Costa, C. T., Horton, D., & Margolis, S. A. (2000). Analysis of anthocyanins in foods by liquid chromatography, liquid chromatography-mass spectrometry and capillary electrophoresis. *Journal of Chromatography A, 881*(1–2), 403–410.

29. Rawdkuen, S., Faseha, A., Benjakul, S., & Kaewprachu, P. (2020). Application of anthocyanins as a color indicator in gelatin films. *Food Bioscience, 36*, 100603.
